# Complexation of CXCL12, FGF-2 and VEGF with Heparin Modulates the Protein Release from Alginate Microbeads

**DOI:** 10.3390/ijms222111666

**Published:** 2021-10-28

**Authors:** Edyta Adrian, Dušana Treľová, Elena Filová, Marta Kumorek, Volodymyr Lobaz, Rafal Poreba, Olga Janoušková, Ognen Pop-Georgievski, Igor Lacík, Dana Kubies

**Affiliations:** 1 Institute of Macromolecular Chemistry, Czech Academy of Sciences, Heyrovsky sq.2, 162 06 Prague, Czech Republic; Edyta.Wawrzynska@vscht.cz (E.A.); martamariakumorek@gmail.com (M.K.); lobaz@imc.cas.cz (V.L.); poreba@imc.cas.cz (R.P.); janouskova324@gmail.com (O.J.); georgievski@imc.cas.cz (O.P.-G.); 2 Department of Chemical Engineering, University of Chemistry and Technology, Technicka 5, 166 28 Prague, Czech Republic; 3 Polymer Institute of the Slovak Academy of Sciences, Dubravska cesta 9, 845 41 Bratislava, Slovakia; mocinecova@gmail.com (D.T.); igor.lacik@savba.sk (I.L.); 4 Department of Biomaterials and Tissue Engineering, Institute of Physiology of the Czech Academy of Sciences, Videnska 1083, 142 20 Prague, Czech Republic; Elena.Filova@fgu.cas.cz; 5 Centre for Advanced Materials Application of the Slovak Academy of Sciences, Dubravska cesta 9, 845 11 Bratislava, Slovakia

**Keywords:** alginate microbeads, heparin, CXCL12, VEGF, FGF-2, protein release, ITC, SPR, bioactivity, HUVECs

## Abstract

Long-term delivery of growth factors and immunomodulatory agents is highly required to support the integrity of tissue in engineering constructs, e.g., formation of vasculature, and to minimize immune response in a recipient. However, for proteins with a net positive charge at the physiological pH, controlled delivery from negatively charged alginate (Alg) platforms is challenging due to electrostatic interactions that can hamper the protein release. In order to regulate such interactions between proteins and the Alg matrix, we propose to complex proteins of interest in this study - CXCL12, FGF-2, VEGF - with polyanionic heparin prior to their encapsulation into Alg microbeads of high content of α-L-guluronic acid units (high-G). This strategy effectively reduced protein interactions with Alg (as shown by model ITC and SPR experiments) and, depending on the protein type, afforded control over the protein release for at least one month. The released proteins retained their in vitro bioactivity: CXCL12 stimulated the migration of Jurkat cells, and FGF-2 and VEGF induced proliferation and maturation of HUVECs. The presence of heparin also intensified protein biological efficiency. The proposed approach for encapsulation of proteins with a positive net charge into high-G Alg hydrogels is promising for controlled long-term protein delivery under in vivo conditions.

## 1. Introduction

Ongoing research in tissue engineering (TE) applications evinces the imperative support of effective integration of implant constructs with the recipient’s tissue using externally supplied signaling molecules, such as growth factors (GFs) and chemokines. These bioactive agents regulate a variety of cellular processes, such as cell migration, proliferation, and differentiation, and they promote neovascularization, angiogenesis, and osteogenesis, and minimize the immune response of the recipient [1,2]. Their encapsulation can prolong protein delivery in local areas, limit the risk of protein proteolysis or denaturation and decrease the risk connected with high-dose toxicity of freely administrated GFs in therapeutic doses [1]. For example, ingrowth of host vasculature within an implanted TE construct of several millimeters and its complete vascularization can take weeks [3]. This process should be supported by prolonged local delivery of pro-angiogenic GFs using the immobilization strategies.

Proteins can be immobilized to different matrices either physically, covalently or by specific binding to the extracellular matrix [1]. According to specific applications, proteins can be released to the place of implantation directly from TE constructs, i.e., from polymer scaffolds, or using delivery carriers, such as particle-based systems. Various natural (e.g., gelatin, collagen, chitosan, alginate, hyaluronic acid, fibrin) and synthetic (e.g., poly-L-lactide, poly(lactic-*co*-glycolic)acid, poly(ethylene argininylaspartate diglyceride), polycaprolactone, polyethylene glycol, polyacrylic acid) polymers have been tested as potential carriers for controlled release of GFs to injured tissues [1,2,4]. Of them, alginate (Alg) has become widely studied for encapsulation of biomolecule factors because of its biocompatibility, low cost and simple gelation under mild conditions [5,6,7,8,9]. Alg is a natural anionic polysaccharide consisting of (1-4)-linked β-D-mannuronic acid (M) and α-L-guluronic acid (G) units that are covalently linked together in different sequences and ratios. Physical gelation occurs in the presence of divalent cations, typically Ca^2+^, when G-blocks are preferentially responsible for the formation of intermolecular crosslinking. The Alg chemical composition (M/G ratio and sequence, G-block length) and molecular weight profoundly influence the physical properties. Alg of a high G content (high-G Alg) forms mechanically stronger, stiffer, and more brittle gels with a higher porosity and bigger pores, while Alg gels of higher M content (high-M Alg) generate comparatively less porous, softer, and more elastic hydrogels. The Alg composition and hydrogel characteristics therefore affect the gel stability and the release rate of encapsulated molecules [6,8,9]. Hydrogels made of high-G Alg exhibit lower swelling, show higher protein adsorption of higher molecular weights, and generally are a preferred matrix for protein entrapment. Hydrogels based on high-M Alg are known for faster protein release [10]. Furthermore, high-G Alg was reported to be less immunogenic to human monocytes and less potent in inducing cytokine production in comparison to high-M Alg [9]. In addition to being studied as particle-based delivery carriers, Alg hydrogels are used as 3D scaffolds, carriers for 3D cell cultures, and components of coatings, or in the field of wound dressings, cell encapsulation, and 3D bioprinting [6,8,9,11,12].

Numerous studies on the encapsulation and release of GFs from Alg carriers have been reported [5,7,10]. For the effective delivery of proteins, a control over protein release from Alg hydrogels is the principal requirement. The release rates of electronegative and neutral proteins under physiological pH are usually rapid, due to the inherent porosity, the absence of attractive electrostatic interactions, and the hydrophilic nature of hydrogels, whereas the negatively charged Alg is expected to electrostatically interact with proteins bearing a net positive charge at physiological pH, i.e., proteins of the p*I* higher than 7.4, which may consequently lower the release rates [5,6,10]. Until now, the interactions of this class of proteins with the Alg matrix were masked through the use of excipients, such as poly-L-lysin, polyacrylic acid, or polyaspartic acid for delivery of transforming growth factor-β (TGF-β, p*I* of 9.5) [13], or bovine serum albumin (BSA) for delivery of vascular endothelial growth factor (VEGF) [14]. While poly-L-lysin and BSA did not significantly affect protein release, complexation of TGF-β with polyacrylic acid before encapsulation into Alg microbeads preserved protein immunoreactivity and resulted in prolonged TGF-β delivery.

A wide range of GFs as well as chemokines are so-called heparin-binding proteins with a strong natural affinity for heparin (Hep) [15]. The binding of these proteins to heparan sulphate or Hep in vivo provides effective conjugation of proteins with corresponding receptors in cell membranes, and thus, triggers their biological activity. This specific non-covalent interaction is frequently exploited to increase the retention of heparin-binding proteins in hydrogel matrices, from which the proteins are released very rapidly. When heparin glycosaminoglycans are covalently bound in such fast-releasing hydrogels, Hep molecules act as non-covalent binding sites for the loaded protein, resulting in significant slowing of the protein release to weeks or even months [16,17,18,19,20,21,22].

The subjects of this study are the heparin-binding proteins of different p*I*s, i.e., CXCL12, fibroblast growth factor 2 (FGF-2), and VEGF, and their long-term release from Alg hydrogels. The chemokine CXCL12 (p*I* of 10.19) is known to regulate the density of inflammatory cells and was shown to participate in angiogenesis and wound-healing processes [23,24]. CXCL12 has been extensively investigated because of its roles in tumor pathogenesis, inflammation processes, autoimmune diseases, and neuroinflammatory disorders [25,26,27]. Locally administered CXCL12 can induce local immune isolation and prolong allo- or xenograft function without systemic immunosuppression; because of this function, CXCL12 has been recently considered a prospective immunomodulatory agent to support or even replace immunosuppression therapy in, for example, islet transplantation [28,29]. FGF-2 (p*I* of 9.45) stimulates cell proliferation, survival, differentiation, migration, and angiogenesis. VEGF (p*I* of 7.02) is involved in vasculogenesis, angiogenesis, physiological vascular homeostasis, differentiation of endothelial progenitor cells into endothelial cells (ECs), vascular repair, and re-endothelialization. VEGF affects the proliferation, differentiation, migration, and survival of various cell types, and inhibits neointima formation [30,31]. Thus, the release of both GFs from various Alg hydrogel matrices has been investigated for TE applications. VEGF and FGF-2 are typically released from Alg carriers for no longer than 12 days, regardless of the content of G or M units [5,7]. To date, however, delivery of the immunomodulant CXCL12 from an Alg matrix has been demonstrated only with microbeads prepared from high-M Alg [32,33], whereas no delivery was observed from high-G Alg microspheres [34], which was attributed to strong charge-mediated interactions between CXCL12 and the high-G Alg matrix.

**Figure 1:**
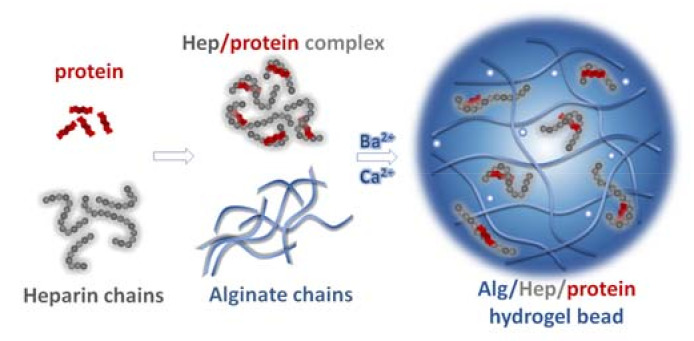
Schematics of preparation of Alg microbeads for delivery of CXCL12, FGF-2, and VEGF proteins that are complexed with Hep prior to their encapsulation.

The hypothesis of this work is that complexing CXCL12, FGF-2, and VEGF with Hep, prior to protein encapsulation into microbeads prepared from high-G Alg, shields binding sites on protein molecules and thus prevents interactions between proteins and the Alg matrix. In this way, the protein release is modulated in a controlled manner. To this end, the high-G Alg microbeads containing a protein complexed with Hep, as schematically depicted in Figure 1, were prepared by an air-stripping method. Human serum albumin (HSA) was also included in the delivery system to protect proteins during microbead preparation as well as during release experiments. Albumin is commonly used for the stabilization of proteins in solutions, leading to preservation of protein biological activity [35]. To evaluate the potential effect of p*I* of the encapsulated protein, the proposed concept was applied to these proteins significantly differing in p*I* values. The interactions between the particular microbead components were studied under model conditions using isothermal titration calorimetry and surface plasmon resonance analysis, the in vitro protein release was determined by an ELISA method, and the bioactivity of the released CXCL12, VEGF, and FGF-2 proteins was evaluated in vitro using human T lymphoma cell line and human umbilical vein endothelial cells (HUVECs).

## 2. Results and Discussion

### 2.1. Preparation and Characterization of Alg Microbeads

The microbeads were prepared from high-G Alg (M/G ratio of 0.59, Table S1) using an air-stripping nozzle instrumental device according to a well-established protocol developed for encapsulation of islets [36,37]. The crosslinking was performed in the presence of Ba^2+^ and Ca^2+^ salts in order to increase the microbead stability [36]. The native Alg microbeads were of a spherical shape and diameter of 0.6–0.7 mm (Figure S4A), and did not rupture under a compression force causing at least 95% deformation (Figure S4B).

**Figure 2:**
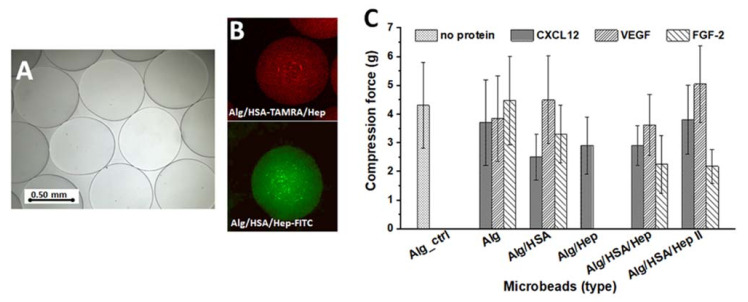
Characterization of alginate (Alg) microbeads containing Heparin (Hep) and HSA as additives. (**A**) A typical morphology of Alg/HSA/Hep microbeads; Hep, HSA, or proteins added into the Alg matrix have no significant effect on the spherical shape and size of the microbeads (light microscopy); (**B**) Visualization of HSA (a red color, stained with TAMRA, red) or Hep (a green color, stained with FITC) spatial distribution within an alginate matrix (Alg/HSA/Hep microbeads, a confocal microscopy); (**C**) Mechanical strength of the prepared microbeads expressed as compression force (in grams) needed for 70% of the microbead deformation. Values represent the means and standard deviation (*n* = 21).

To modulate the release of CXCL12, VEGF, or FGF-2, we loaded the proteins as a complexed mixture with Hep into the Alg matrix. In addition, HSA was used as a protective additive. The composition of each solution used for microbead preparation is presented in Table 1 (see Section 3). An excessively high content of any additive can affect the crosslinking of the Alg chains, causing the hydrogel network to become unstable and to disintegrate. Indeed, more than 0.14 wt. % of additives (such as polyacrylic acid or poly-L-lysine) in a 0.5 wt. % Alg solution prevented the effective crosslinking process [13]. In our work, the content of all the additives was lower than 0.04 wt. %, and the additives did not influence the microbead shape (Figure 2A) or size. Importantly, Hep and HSA were almost homogeneously dispersed within the Alg matrix (Figure 2B). As the additive content was very low, no remarkable changes in the mechanical properties of the microbeads were expected. The addition of protein alone did not affect the mechanical stability, and the compression force tended to slightly decrease only for microbeads containing HSA and Hep, especially in the case of microbeads loaded with CXCL12 or FGF-2 (Figure 2C). During the incubation in PBS for one month, no disintegration of the microbeads was observed (data not shown).

### 2.2. Interactions of Hep, HSA, and Proteins with Alg in the Solution and in the Form of Crosslinked Thin Layers

Alginate and heparin are negatively charged polyelectrolytes with a p*K*_a_ range of 3.40–4.41 [8] and 2.79–3.13 [38], respectively. On the other hand, the CXCL12 and FGF-2 proteins are positively charged at physiological pH with a calculated p*I* of 10.19 and 9.45, respectively. Therefore, interactions between the components based on electrostatic attraction forces were expected to affect protein release from the Alg matrix. In contrast, VEGF with a p*I* of 7.02 was not expected to electrostatically interact with Alg in any significant way.

**Figure 3:**
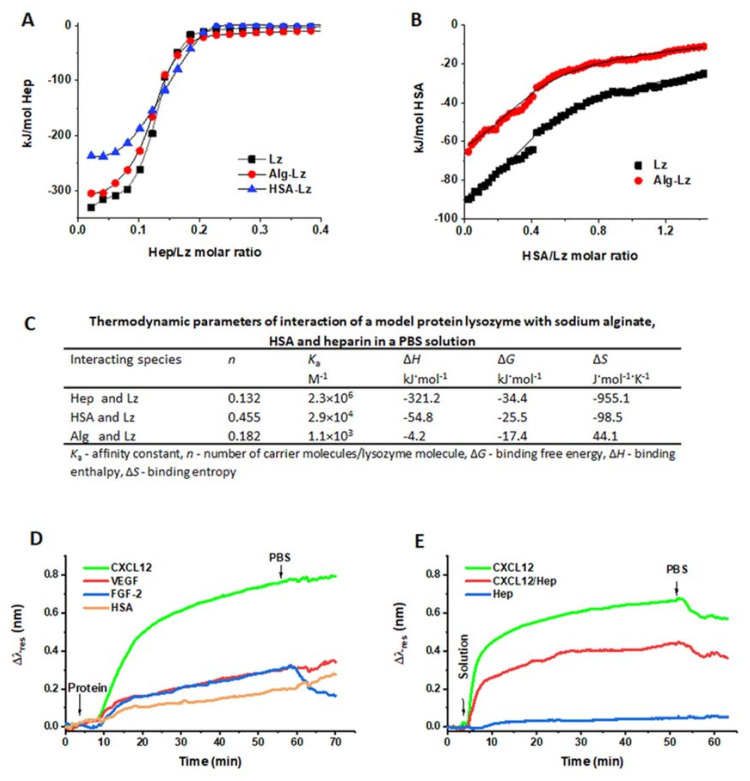
T Interactions between the components of Alg-based microbeads studied by ITC and SPR analysis. (**A**) Titration isotherms of heparin to a model protein lysozyme (Lz) and to Alg-Lz and HSA-Lz complexes in PBS (ITC); (**B**) Titration isotherms of the HSA to Lz and the Alg-Lz complexes in PBS (ITC); (**C**) Thermodynamic parameters of the interactions of Lz with Alg, HSA, and heparin (ITC); (**D**) Binding of CXCL12, VEGF, FGF-2, and HSA to the crosslinked Alg layer (SPR); (**E**) Binding of CXCL12, Hep, and a CXCL12/Hep mixture to the crosslinked Alg layer (SPR). Arrows show the replacement of the solutions. The data for the shift of resonance wavelength, Δ*λ*_res_, of the control PBS were subtracted from the data obtained for the tested samples. The slight drift in the baseline (observed in all experiments) indicates that some continuous changes in the structure of the Alg layer likely occurred upon the exposure of the SPR chip to PBS.

First, we used lysozyme (Lz) with a p*I* of 10.7 [39] as a model protein for evaluation by isothermal titration calorimetry. Alg, Hep, and HSA (with a p*I* of 4.7, [40]) did not interact with each other in a PBS solution (Figure S5A). The binding of Lz to Alg was not measurable with direct ITC titration, although it was visible with the naked eye as the formation of a turbid colloid (Figure S5B). In contrast, Hep and HSA demonstrated a strong exothermic reaction with Lz (Figure 3A,B, black data). The titration of Hep to preformed Alg-Lz and HSA-Lz complexes showed less exothermic and weaker interactions of the complexed Lz in comparison with the free Lz (Figure 3A,B). This experimental arrangement also allowed indirect evaluation of thermodynamic parameters for Lz binding to Alg (see the SI, Part S2.4 for a detailed description). The calculated thermodynamic parameters for the Hep-Lz, HSA-Lz and Alg-Lz complexes (Figure 3C) indicate that all three polymers were able to bind Lz at physiological pH (Δ*G* < 0), binding was exothermic (Δ*H* < 0), and binding strength (in terms of *K*_a_) decreased in the order Hep > HSA > Alg. For Hep and HSA, the binding of Lz was accompanied by an entropy decrease (Δ*S* < 0), which is often observed for exothermic interactions through Coulomb forces. The weak binding of Lz to Alg (a weak acid) is rather driven by entropy (Δ*S* > 0) than by a strong attraction of countercharges.

Since ITC measurement is not a convenient method for following interactions between high-cost proteins and Alg, we also performed an SPR analysis to follow the interactions between CXCL12, FGF-2, VEGF, and Alg chains as well as the Alg chain interactions with Hep and HSA (Figure 3D,E). We immobilized the crosslinked Alg layer on a SPR chip, which can be considered as a model system for testing the interactions of various compounds with a crosslinked Alg hydrogel matrix. Such a crosslinked layer approximates the hydrogel structure that forms Alg microbeads. Although the ITC evaluation did not reveal any interactions between HSA and Alg in the PBS solution (Figure S5A), Figure 3D shows that a certain amount of HSA bound to the Alg layer. This behavior can be attributed to hydrogen bonding and hydrophobic interactions between these two components, which are presumed to play important roles, for example, in BSA/Alg binding [41,42]. Figure 3D also demonstrates that CXCL12 was deposited on the Alg layer at a higher level than the other tested proteins, which was most likely a result of CXCL12 having the highest p*I*. Nevertheless, although there is a significant difference in the p*I* of VEGF and FGF-2, the binding of these proteins to the model crosslinked Alg layer was, surprisingly, almost the same. Therefore, we can assume that hydrogen bonding interactions also contribute to the binding of CXCL12, FGF-2, and VEGF to Alg, as it was proposed for BSA [41]. Additional studies are required to analyze this assumption.

Figure 3E demonstrates no binding of Hep to the model Alg hydrogel layer, which corresponds with no interactions being established between these two components in solution, as determined by ITC (Figure S5A). Figure 3E also shows that less matter was deposited by the CXCL12/Hep co-solution than by the pure CXCL12 solution. Thus, in this model SPR experimental setup, the complexation of CXCL12 with Hep (here, the CXCL12/Hep ratio corresponds with the CXCL12/Hep ratio used for microbead preparation) partially restricted the interactions of CXCL12 with Alg.

### 2.3. Release of CXCL12, FGF-2, and VEGF from Alg Microbeads

**Figure 4:**
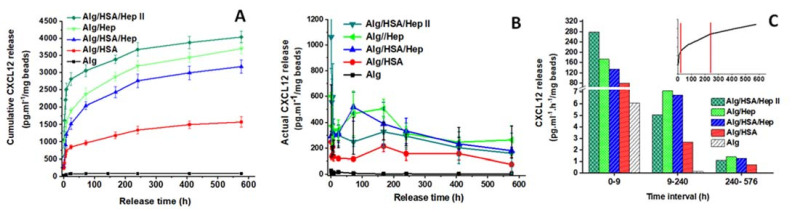
Effect of Hep HSA and additives on the CXCL12 release from Alg microbeads. (**A**) Cumulative CXCL12 release; (**B**) Actual CXCL12 release at particular time intervals; (**C**) Actual CXCL12 release recalculated for one hour within three selected time intervals; time intervals were selected according to the slope of the plotted curve (the inset). Values represent the mean and standard deviation (*n* at least 3). (The protein released in PBS upon the addition of 0.1 wt. % BSA and 0.02 wt. % NaN_3_; the ELISA determinations; microbead compositions are presented in Table 1).

A preliminary study was performed using CXCL12-loaded Alg microbeads (Table 1). Of the proteins tested, **CXCL12** had the highest p*I* of 10.19, suggesting that nonspecific interactions presumed to be of electrostatic origin between CXCL12 and Alg may slow or even block CXCL12 diffusion through the Alg hydrogel network. Indeed, almost no CXCL12 was released from the pure Alg samples (Figure 4A,B, black curve). In contrast, Alg/HSA/Hep, Alg/HSA/HepII, Alg/Hep, and Alg/HSA microbeads delivered the protein for at least four weeks. The course of cumulative release indicated an initial fast release in the first nine hours, followed by a decreased release for two weeks and then a slow sustained delivery for the next two weeks until the end of the study (Figure 4A). Unexpectedly, the Alg/HSA microbeads delivered a 100-fold higher amount of CXCL12 than the pure Alg microbeads during the first nine hours (Figure 4A, red curve), followed by almost sustained release of approximately 150 pg⋅mL^−1^/mg microbeads until the end of the study (Figure 4B). The release from the Hep-containing microbeads was more profound, and increased with an increasing Hep amount (Figure 4A); the Alg/HSA/HepII microbeads with twice the Hep content released twice as much CXCL12 (up to 2800 pg⋅mL^−1^/mg microbeads) than the Alg/HSA/Hep microbeads during 24 h. Thereafter, the release rate was comparable in both samples for four weeks (Figure 4B).

Figure 4C presents a more illustrative description of the effect of additives on the CXCL12 release. The course of the curves (Figure 4C, inset) indicates a triphasic pattern with different curve slopes, which correspond to a nearly zero-order release (i.e., 0–9 h, 9–240 h and 240–576 h time regions). For these time intervals, the actual release data were recalculated as “the protein amount released per hour from one milligram of microbeads”. We assume that the highest CXCL12 release of 280 pg⋅mL^−1^⋅h^−1^/mg microbeads from the Alg/HSA/HepII sample during the first nine hours was related to the Hep content being the greatest in these microbeads. All release rates decreased over the next two weeks (9–240 h); the pure Alg microbeads released no protein, and the Hep-containing microbeads released twice as much CXCL12 (5–7.5 pg⋅mL^−1^⋅h^−1^/mg microbeads) as the Alg/HSA microbeads. Finally, a diminished but steady release between 1 and 1.5 pg⋅mL^−1^⋅h^−1^/mg microbeads continued until the end of the experiment.

**Figure 5:**
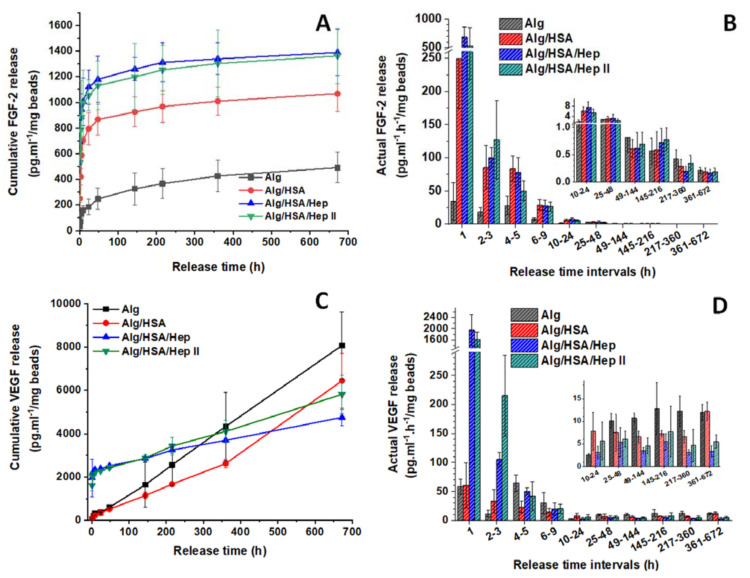
Effect of HSA and Hep additives on the release of FGF-2 (**A**,**B**) and VEGF (**C**,**D**) from Alg microbeads: data for the cumulative release (**A**,**C**), data for the actual release recalculated for one hour in particular time intervals (**B**,**C**). Values represent the mean and standard deviation (*n* at least 3). (The protein released in PBS upon the addition of 0.1 wt. % BSA and 0.02 wt. % NaN_3_; the ELISA determinations).

The cumulative release and the actual release calculated for one hour of **FGF-2** (p*I* = 9.45) are shown in Figure 5A,B. In contrast to CXCL12, FGF-2 is not fully retained in the pure Alg hydrogel, probably because the p*I* of FGF-2 is lower than that of CXCL12. Therefore, weaker nonspecific electrostatic interactions are assumed to be established between FGF-2 and the Alg chains. All curve patterns exhibited an initial release followed by a slower release for as many as two days, and a low but gradual FGF-2 delivery of less than 1.0 pg⋅mL^−1^⋅h^−1^/mg microbeads until the end of the study. Both Hep and HSA promoted the FGF-2 release, especially within 48 h (about 80% of the total release amount for the particular samples). After four weeks, the Alg/HSA and Hep-containing microbeads released four- and six-fold higher amounts of FGF-2 than the Alg microbeads. Opposed to the CXCL12-loaded microbeads, no dependence on the Hep content was observed.

The release of **VEGF** (p*I* = 7.02) exhibited different characteristics (Figure 5C,D) than CXCL12 and FGF-2. The highest 4-week cumulated amount of VEGF was released from the pure Alg microbeads. There was no considerable initial phase detected, and the release was linear with a constant amount of approximately 10 pg⋅mL^−1^⋅h^−1^/mg microbeads released over four weeks. The Hep-containing microbeads released an approximately 40-fold higher amount of protein than that released from the pure Alg microbeads during 24 h. Then, the release slowed and remained sustained (approximately 5.0 pg⋅mL^−1^⋅h^−1^/mg microbeads) until the end of the study. In contrast to the CXCL12 and FGF-2, the presence of HSA did not affect the VEGF release profile markedly.

**Delivery of pure proteins.** To date, only a limited number of reports on **CXCL12** release from Alg matrices have been published [28,32,33]. The high-M Alg microbeads prepared by an electrostatic droplet generator released CXCL12 for 5 days, and then, the proposed retention of CXCL12 in the hydrogel resulted in minimal release for the next 20 days [33]. In another study, the low but sustained CXCL12 release of 0.18 ng⋅mL^−1^⋅h^−1^/mL microbeads was reported for 25 days [32]. These results are in contrast with the negligible CXCL12 release from high-G Alg microbeads prepared in our study (Figure 4) and no release from high-G Alg microspheres published by Wang et al. [34]. Only a very limited CXCL12 release of 1% was observed after 5 h compared to the increased release of CCL21, CCL19 and CXCL10 chemokines. The different diffusivity of the chemokines from Alg was attributed to the impact of the net surface charge of the proteins on the charge-mediated interactions between the proteins and Alg matrix, which seem to dominate the release behavior [34]. Thus, it is apparent that the preparation conditions in terms of Alg type, crosslinking process and technology settings dictate the character of the CXCL12 release from different types of Alg hydrogel carriers.

According to the literature, most Alg particles or scaffolds are prepared from high-M Alg and crosslinked by Ca^2+^ cations. Typical release profiles of **VEGF** from Alg carriers exhibit an initial fast release during the first few hours, followed by a deceleration for 2–4 days and then usually level off to a plateau with minimal or no release. These particles or scaffolds, prepared by needle extrusion [14], as in our study, emulsification techniques [43,44], spray-drying processes [45] or by crosslinking in bulk [46,47], delivered proteins between five and ten days. No significant effect of the Alg type on protein retention and release was observed, although electrostatically based affinity interactions between VEGF and Alg mediated predominantly by M-containing blocks were hypothesized [43]. In contrast, the high-G Alg microbeads crosslinked with Ca^2+^/Ba^2+^ ions evaluated in our study released VEGF without an initial high release in a zero-order kinetic pattern for at least four weeks (Figure 5C). Comparable VEGF release profiles up to 21 to 40 days were reported only for high-M Alg microparticles or high-G oxidized-Alg/Alg bulk hydrogels, where the release was attributed to slow continuous degradation of the hydrogel matrix (described as disintegration) in the releasing medium [48,49,50,51]. The microbeads tested in our study were stable in PBS throughout the release experiments.

**FGF-2** or FGF-1 were also usually released for no longer than 12 days from different types of Alg hydrogels, i.e., microbeads prepared by needle extrusion or air stripping [47,52], bulk scaffolds [53,54], nanoparticles prepared from pegylated Alg using the emulsion technique [55], and microgels prepared by the microfluidic approach [56]. High-M Alg delivery systems delivered FGF-2 mostly within a few hours, then a minimal release of 1 to 5% was detected [52,53]. In our study, only 50% of the total FGF-2 amount was released within 48 h, and then the release gradually continued for one month (Figure 5A). The short-term release for several days approaching sustained release profiles has been reported only for highly crosslinked high-G Alg particles [47,55,56].

Different release profiles of CXCL12, VEGF, and FGF-2 (Figure 4 and Figure 5) suggest specific interactions of particular proteins with the pure Alg hydrogel. The total amount of the released protein decreased in the order VEGF > FGF-2 > CXCL12. Additionaly, the SPR analysis showed the highest deposition of CXCL12 on the model Alg layer (Figure 3D). The observed trends correlate with the increasing p*I* of the proteins in the same order of release, suggesting a strong contribution of electrostatic interactions to protein/Alg binding, as has also been proposed in the literature [34,57,58]. However, the amounts of VEGF and FGF-2 deposited on the model Alg layers were similar (Figure 3D), even though the release profiles differed. In addition, the p*I* of FGF-2 and CXCL12 is not markedly different, but no CXCL12 was released from pure Alg microbeads, in contrast to the evident release of FGF-2. These observations imply that interactions other than ionic interactions must also be considered to play a role in the release of these proteins, such as hydrogen bonding and hydrophobic and van der Waals interactions [41,42,59,60]. Furthermore, the diffusion of proteins through the hydrogel network is also expected to depend on the protein diameter with respect to the mesh diameter. We can assume that the mesh size of the microbeads prepared in our work did not differ significantly from the average mesh size of high-G Alg hydrogel cylinders crosslinked with Ba^2+^ cations [61], which was determined to be of 10–25 nm by rheological and swelling measurements. Additionally, high-G Alg microbeads crosslinked with Ca^2+^/Ba^2+^ cations exhibited a molecular weight cutoff of 350 kDa for globular proteins, which corresponds to a mesh size of approximately 12 nm [37]. As the hydrodynamic radius of FGF-2, VEGF, and CXCL12 is not greater than 3 nm [62], the protein size cannot be considered a factor that significantly affects the diffusion of proteins through the hydrogel matrix.

**Delivery of proteins complexed with Hep and HSA.** The growth factor TGF-β with the p*I* of 9.5, which is comparable to the p*I* of CXCL12 or FGF-2, was not released from Alg microbeads regardless of the G- or M-unit content [13]. However, the release was detected when polyacrylic or polyaspartic acid was used as an additive for masking the interaction between the protein and Alg chains. In our work, we tested the polyanion Hep as a masking agent to affect the release of heparin-binding GFs with a high p*I*, such as CXCL12, FGF-2, and VEGF with the p*I* close to physiological pH, from high-G Alg hydrogels. The concept of shielding protein binding sites was indirectly proven by following the interactions between the microbead components and the model protein Lz using ITC. The reverse titration of the Alg-Lz complex in PBS with Hep showed that Lz preferentially complexes with Hep (K_a_ of 2.3 × 10^6^ M^−1^) rather than Alg (*K*_a_ of 1.1 × 10^3^ M^−1^) (Figure 3C). Because the *K*_a_ values of FGF-2, VEGF, and CXCL12 with Hep were determined to be 1.68 × 10^7^ M^−1^, 8.14 × 10^5^ M^−1^, and 3.2 × 10^7^ M^−1^ [63,64], respectively, it can be expected that these proteins would also preferentially complex with Hep. Furthermore, a lower amount of CXCL12-Hep complex than pure CXCL12 was deposited on the model Alg layer (Figure 3E), suggesting a partial blocking of the interactions between Alg and CXCL12 complexed with Hep.

Consistent with the ITC and SPR data, the complexation of CXCL12 with Hep resulted in the release of a 500-fold higher amount of CXCL12 in one month, in contrast to the negligible protein release from the pure Alg microbeads (Figure 4). Assuming that the type and size of the microbeads prepared by us and by Chen et al. [32] are comparable (in our study, 1 mL of Alg microbeads corresponded to 433 mg of wet weight of microbeads), the actual release of 0.18 ng⋅mL^−1^⋅h^−1^/mL microbeads [32] can be recalculated approximately as 0.42 pg⋅mL^−1^⋅h^−1^/mg microbeads. So, the actual CXCL12 release rates per hour in our study (Figure 4C) were approximately one order of magnitude higher than that reported for high-M Alg microbeads [32]. Although not as significant as with CXCL12, the 4-week total amount of FGF-2 released from the Hep-containing microbeads was six-fold higher than that released from the pure Alg microbeads (Figure 5), probably because the p*I* of FGF-2 is lower than that of CXCL12. As for VEGF, which had the lowest p*I*, the Hep-containing microbeads released a significantly higher dose than the Alg microbeads only within the first 24 h, and then the release remained sustained for the following weeks (Figure 5C). Until now, the natural affinity of heparin-binding proteins for Hep was employed to prevent an extremely rapid release of VEGF and FGF-2 from some types of Alg hydrogels. In these cases, the use of Hep was aimed at increasing protein retention in covalently crosslinked Alg/Hep hydrogel matrices, but the release was extended to only six to fifteen days [22,53].

The addition of HSA to the microbeads increased protein release as well. The *K*_a_ of the model protein Lz with HSA was determined to be one order of magnitude higher than that of Lz with Alg (Figure 3C). Since the p*I* of CXCL12 and FGF-2 is similar to the p*I* of Lz, we can suppose that both proteins also complex preferentially with albumin; therefore, protein binding to Alg is at least partially limited. The increased release of CXCL12 and FGF-2 from the Alg/HSA microbeads (Figure 4A and Figure 5A), although not as significant as that from the Hep-containing microbeads, corresponds with this assumption. In contrast, no remarkable effect of BSA on VEGF release was observed [14], which was also confirmed in our experiments (Figure 5C).

### 2.4. In Vitro Biological Evaluation

#### 2.4.1. Effect of CXCL12 Released from Alg Microbeads on Migration of Jurkat Cells

In TE applications or cell transplantations, the homeostatic chemokine CXCL12 has recently been studied mainly due to its influence on the recipient’s immune response. CXCL12 promotes the migration of a wide range of cells, such as lymphocytes, macrophages, monocytes, hematopoietic progenitor and stem cells, ECs, and cancer cells [24,25,26,27]. Thus, the in vitro cell migration assay is frequently used to verify the CXCL12 biological activity. Among four cell types tested, i.e., two mouse macrophage cell lines, a mouse mammary carcinoma cell line, and a human T lymphoma cell line (Jurkat cells), the Jurkat cells exhibited the highest expression of the CDCR4 receptor for CXCL12 (Figure S6A), and, therefore, these cells were used for all the follow-up experiments.

**Figure 6:**
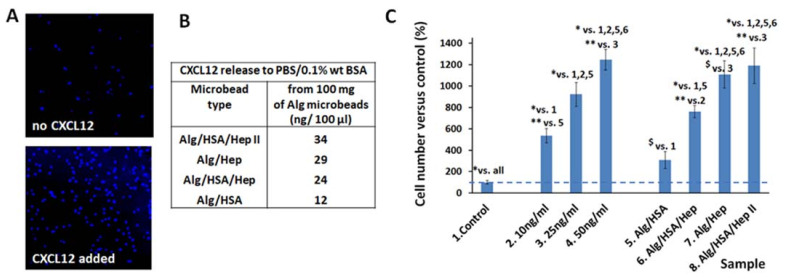
Bioactivity of CXCL12 released from the Alg microbeads: (**A**) Visualization of Jurkat cells that migrated in a CXCL12-free cultivation medium and upon stimulation with the CXCL12 released from the microbeads (a confocal microscopy); (**B**) The calculated amount of CXCL12 released from the microbeads into 100 µL of a PBS/0.1 wt. % BSA solution used for in vitro experiments (an ELISA determination); (**C**) Migration of Jurkat cells stimulated by CXCL12 released from the microbeads and by a free CXCL12 added into the cultivation medium (positive controls). Values represent the number of migrated cells expressed as % to the control (the means and standard deviation, *n* = 5). For the microbead composition see Table 1. One-way analysis of variance, the Student–Newman–Keuls method was used for statistical analysis. Indicators above the columns show statistical differences compared to the samples identified with number indicated. The data is expressed as the mean ± SD, * refers to *p* < 0.001, ** to *p* < 0.01, $ to *p* < 0.05.

To achieve the appropriate cell migration response, as illustrated in Figure 6A, we optimized the experimental conditions of a Boyden chamber assay in terms of the number of seeded cells, the time of migration and the volume of the releasing solution (PBS with 0.1 wt. % BSA) that should be added to the cultivation medium without undesirable effects on the cell migration (Figure S6B,C). The addition of 10 vol. % of PBS allowed for sufficient cell migration of 75% compared to the migration in the medium without PBS added. At the same time, the added volume contained a sufficient amount of CXCL12 to affect the cell migration (Figure 6B). Figure 6C presents the effect of the delivered CXCL12 on the migration of Jurkat cells, which is expressed as % versus the negative control, which was set as 100%. Jurkat cells migrated noticeably more than in the control, even though a low amount of CXCL12 was present in the cultivation medium corresponding to the Alg/HSA sample. In addition, the increase in the cell migration rate correlated with an increase in the CXCL12 amount added to the cultivation medium (Figure 6B) and reached more than 1000% of the control in the Alg/HSA/HepII and Alg/Hep samples. These samples exhibited the highest CXCL12 release profile (Figure 4). We can conclude that CXCL12 is released from the prepared Alg-based carrier systems in sufficient quantities to affect cell response and retains its bioactivity.

#### 2.4.2. Effect of FGF-2 and VEGF Released from Alg Microbeads on HUVEC Proliferation and Differentiation

To study the effect of the released GFs on HUVECs, the cultivation medium was used without the addition or with a partial addition of GFs supplied by the manufacturer. Fully supplemented medium was used as a positive control. The composition of each cultivation medium is shown in Table 2 (see Section 3).

##### Cell Adhesion and Proliferation

**Figure 7:**
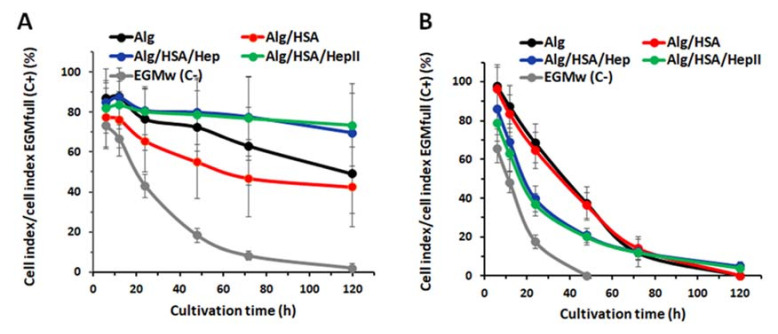
Relative cell proliferation expressed as “Cell index/Cell index in EGMfull” ratio for HUVEC cells during a 5-day culture when the cultivation medium was supplemented with FGF-2. (**A**) or VEGF (**B**) released from the microbeads. Growth factors were released from Alg, Alg/HSA, Alg/HSA/Hep and Alg/HSA/HepII microbeads to PBS/0.1 wt. % BSA. Cell index in EGMfull medium (a positive control) was set at 100%. EGMw(C-) is a cultivation medium with no growth factors added and served as a negative control. The cell index is the parameter providing the status of the cells, including their cell number, viability and morphology.

In this part of the in vitro study, the cells were cultivated with GFs released from the Alg microbeads into PBS with the addition of 0.1 wt. % BSA for 5 days. The effect of FGF-2 and VEGF on cell adhesion and proliferation was analyzed by real-time cell analysis (RTCA). The cell index values were determined for 1-, 2-, 3- and 5-day cultures, with the data presented in Figure S7, including statistical analysis. To show the trends more clearly, the cell index data were expressed as % relative to the positive control (set at 100%), as shown in Figure 7.

In the case of microbeads releasing **FGF-2**, on day 1 after seeding (Figure S7A), FGF-2 released from all types of microbeads induced lower cell index (i.e., both cell adhesion/spreading and cell proliferation) than the positive control medium (EGMfull medium), but a significantly higher cell index than the cell index in the negative control medium (EGMw medium). The cell index for the Alg/HSA/Hep, Alg/HSA/HepII and Alg samples was approximately 80%, and for the Alg/HSA sample, approximately 65% of the positive control (Figure 7A). Cell adhesion is the predominant process during the first day of cell culture. In addition, FGF-2 has been observed to inhibit adhesion of ECs [65]. This effect may contribute to comparable cell indexes of the Hep-containing and pure Alg samples, although the Alg microbeads released a lower FGF-2 amount. A similar trend was observed on day 2, except for the negative control, as the EGMw medium no longer supported HUVECs proliferation (Figure S7B), and the cell index reached only 18% of the positive control (Figure 7A). The cell index increased in all samples except for the EGMw from day 1, which indicates that the amount of delivered FGF-2 was sufficient to maintain cell proliferation. Furthermore, from day 3, we observed a more favorable effect in the eluates from both Hep-containing microbeads with a pronounced increase in the cell index with culture time when compared to the Alg or Alg/HSA microbeads (Figure S7C,D). These results are in agreement with findings that heparin regulates binding of FGF-2 to its receptor, encourages complex formation, and triggers mitogenic signaling [66]. On day 5, the cell index in the Alg/HSA/Hep and Alg/HSA/HepII samples was approximately 70% of the positive control, while proliferation in the Alg or Alg/HSA samples reached only approximately 45%, and the cells did not grow at all in the EGMw (Figure 7A).

When microbeads were loaded with **VEGF**, on day 1 after seeding, the cell index was maximal in the EGMfull medium, lower in the EGMw medium containing microbead eluates, and lowest in the EGMw medium (Figure S7A’). The cell index of HUVECs in the Alg and Alg/HSA samples was 68% of the positive control and reached 40% in the sampes with the eluates from the microbeads containing heparin (Figure 7B). On day 2, the cell index decreased in all tested samples and even reached negative values in the EGMw medium, showing the cell death in the negative controls (Figure S7B’ and Figure 7). The cell index continued to decline over time. However, the Hep-containing microbeads were the only microbeads, which prevented the cell detachment/death until day 5 (Figure S7C’,D’ and Figure 7).

Endothelial cells must be cultured in a high-quality EGMfull medium. The addition of PBS with/without the release eluate into EGMw led to a decrease in the cell culture medium nutrient level, rendering the medium inadequate for HUVEC survival. Both FGF-2 and VEGF regulate cell proliferation, migration and differentiation, and together with angiopoietin, these GFs are essential for initiating angiogenesis [30,31]. However, FGF-2 evinced an inhibitory effect on HUVEC adhesion but a strong enhancement of cell proliferation. In contrast, VEGF affected cell proliferation only moderately, with no negative effect on HUVEC adhesion [65]. These data correlate with our findings on proliferation in a nutrient-deprived culture medium, where FGF-2 in the eluates exhibited a stronger effect on HUVEC proliferation than VEGF, and the cells exposed to FGF-2 probably developed more cell–cell contacts that prevented the cell death.

##### Cell Morphology and Expression of Specific Markers of Differentiation

**Figure 8:**
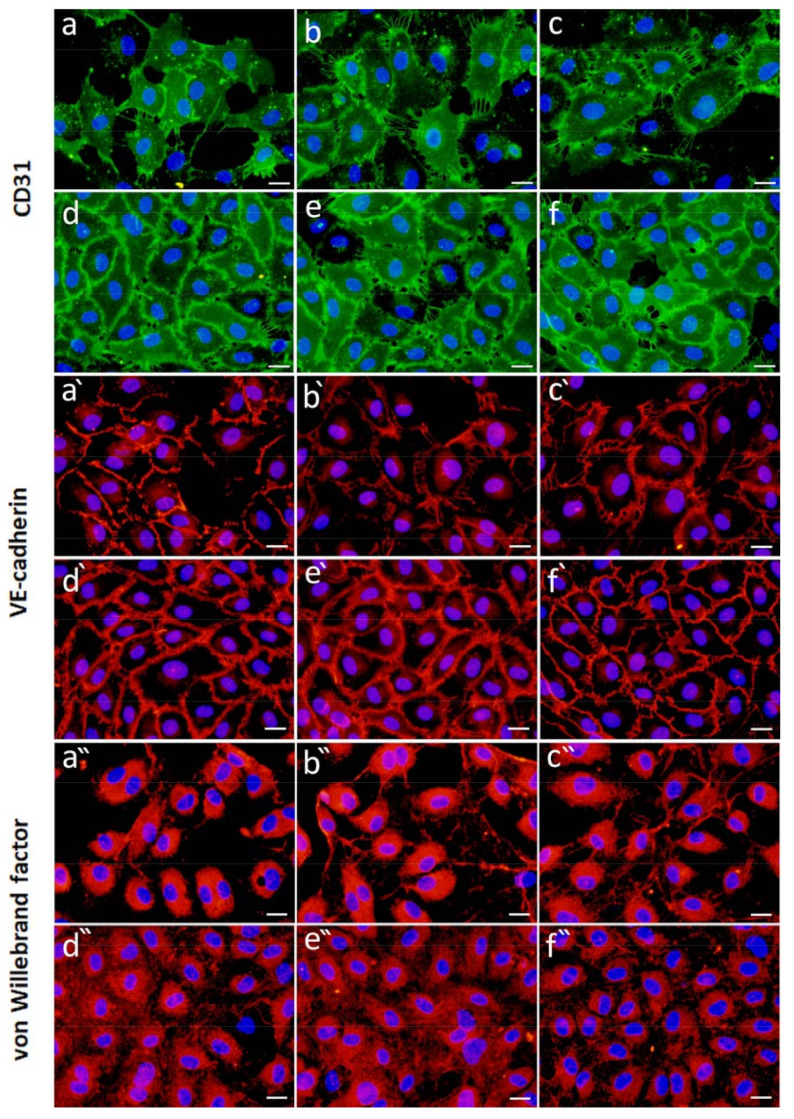
HUVECs cultured in the presence of alginate-based microbeads releasing FGF-2. Representative images of immunofluorescence staining of CD31 (upper raw), VE-cadherin (middle raw), and von Willebrand factor (bottom raw) in HUVECs 5 days after seeding. HUVECs were cultured in the EGMm medium (the negative control; (**a**,**a’**,**a”**)), in the EGMm medium supplemented with the FGF-2-loaded microbeads, i.e., Alg (**b**,**b’**,**b”**), Alg/HSA (**c**,**c’**,**c”**), Alg/HSA/Hep (**d**,**d’**,**d”**), and Alg/HSA/HepII (**e**,**e’**,**e”**), and in the EGMfull medium (the positive control; (**f**,**f’**,**f”**)). An epifluorescence microscope with 40 × objective (scale bar = 20 µm) was used. (The composition of each medium is presented in Table 2).

**Figure 9:**
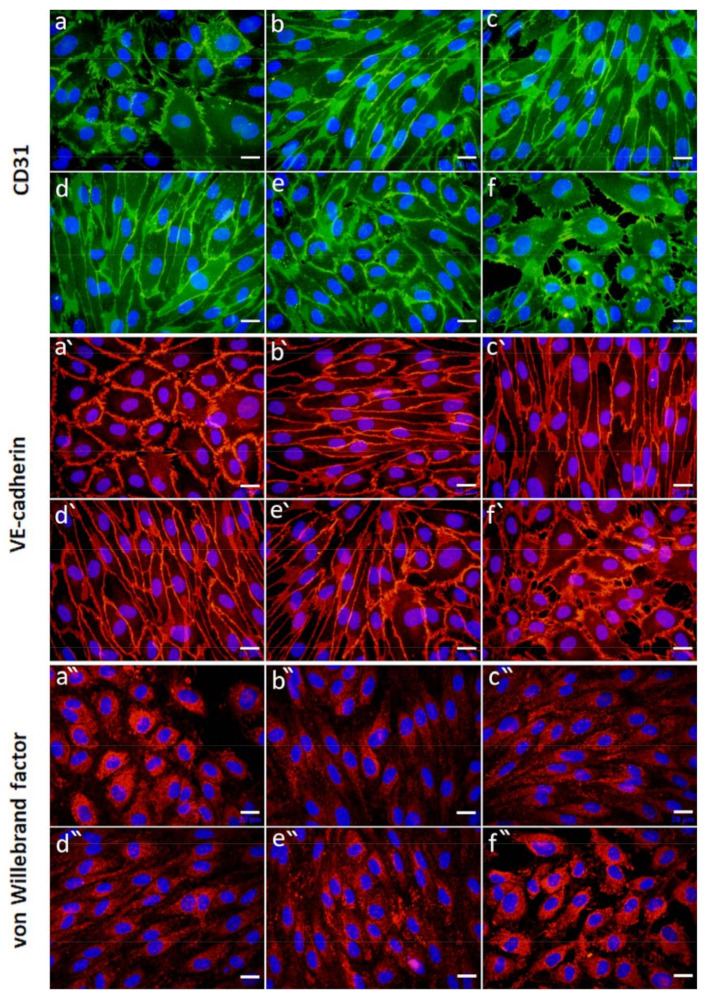
HUVECs cultured in the presence of Alg-based microbeads releasing VEGF. Representative images of immunofluorescence staining of CD31 (upper raw), VE-cadherin (middle raw), and von Willebrand factor (bottom raw) in HUVECs 5 days after seeding. HUVECS were cultured in the EGMm medium (the negative control; (**a**,**a’**,**a”**)), the EGMm medium supplemented with the VEGF-loaded microbeads, i.e., Alg (**b**,**b’**,**b”**), Alg/HSA (**c**,**c’**,**c”**), Alg/HSA/Hep (**d**,**d’**,**d”**), and Alg/HSA/HepII (**e**,**e’**,**e”**), and in the EGMfull medium (the positive control; (**f**,**f’**,**f”**)). An epifluorescence microscope with 40 × objective (scale bar = 20 µm) was used. (The composition of each medium is presented in Table 2).

In this part of the in vitro study, the cells were cultivated directly with the microbeads releasing GFs. The composition of each cultivation medium is shown in Table 2. Representative data from immunofluorescence staining of CD31, VE-cadherin and von Willebrand factor (vWF) are presented in Figure 8 and Figure 9.

In the experiment with microbeads releasing **FGF-2** (Figure 8), HUVECs formed a confluent layer when cultured with Hep-containing microbeads and in the positive control (EGMfull medium). In these samples, CD31 staining was more apparent and regularly distributed (Figure 8d–f) than in the Alg and Alg/HSA samples and in the negative control (EGMm medium), in which CD-31 was more unevenly distributed (Figure 8a–c). However, the intensity of CD31 staining per cell was comparable for all samples (Figure S8A). VE-cadherin staining was intense and localized in cell borders in the Hep-containing samples and in the positive control (Figure 8d’–f’). While VE-cadherin was developed only partially with a more diffuse localization in the Alg and Alg/HSA samples and in the negative control (Figure 8a’–c’), indicating poor maturation of HUVECs in these samples. The highest intensity of VE-cadherin was observed for the Alg/HSA/HepII sample (Figure S8B). Figure 8a”–f” show intense intracellular staining of vWF in HUVECs cultured with the Alg and Alg/HSA microbeads and in the negative control, but the extracellular staining was negligible. Additionally, the staining intensity was the highest in these samples (Figure S8C). On the other hand, the cells cultured with the Hep-containing microbeads and in the EGMfull medium were positively stained with a weaker intensity, but the extracellular localization of the signal was evident (Figure 8d”–f”), suggesting a faster synthesis of vWF in HUVECs.

Immunofluorescence staining of HUVECs cultured with microbeads releasing **VEGF** showed a different cell morphology. The cells were mostly elongated and reached higher densities when cultivated with the Alg, Alg/HSA, and Alg/HSA/Hep microbeads (Figure 9b–d). In contrast, more cobblestone-like cell morphology and a lower cell density were observed in both controls (EGMm and EGMfull) and partially in the Alg/HSA/HepII sample (Figure 9a,e,f). CD31 was expressed by the cells in all samples comparably, and the intensity was slightly higher in the Alg/HSA/Hep than in the Alg/HSA/HepII, Alg, and EGMfull samples (Figure 9 and Figure S8D). All HUVECs were positively stained for VE-cadherin (Figure 9a’–f’ and Figure S8E), and a strong signal was localized in the cell border. VE-cadherin-mediated connections seemed to be better developed in dense cell cultures (Figure 9b’–e’). In contrast, the cells were just developing them in both EGMm and EGMfull controls, as they did not reach confluence by day 5 (Figure 9a’,f’). The immunofluorescence staining of vWF was stronger in the Alg/HSA/HepII samples than in all other Alg-based microbeads (Figure S8F), and the signal was localized mainly intracellularly (Figure 9a”,f”). The increased expression and intracellular localization of vWF seem to correlate with the presence of proliferating cells under confluency. In summary, in the Hep-containing samples releasing FGF-2, we observed lower cell production of vWF, which was secreted into the extracellular matrix. The cells cultured with the microbeads releasing VEGF reached confluency in all the samples and produced more vWF in the Alg/HSA/HepII without its significant deposition in the extracellular matrix.

The preserved biological activity of released therapeutic substances is an essential property of delivery systems that is necessary for their successful application. Overall, all proteins released from the prepared Alg microbeads affected the cell behavior in vitro. CXCL12 stimulated the migration of human T lymphoma cells in a dose-dependent manner (Figure 6), and FGF-2 and VEGF showed a positive effect on HUVEC proliferation and maturation under otherwise very unfavorable cell growth conditions, i.e., in low serum-content and growth factor-deprived media (Figure 7, Figure 8 and Figure 9). The cells cultivated with VEGF-loaded microbeads reached higher densities, were elongated, and expressed more VE-cadherin than cells cultivated in EGMfull medium (Figure 9). VEGF is known to stimulate sprouting, and was also proven to induce elongation of ECs when administered exogenously. ECs elongation appears to be an important process that drives angiogenesis [67,68]. In addition, rapid formation and maintenance of a confluent layer of ECs in a vessel lumen are necessary to prevent blood coagulation, and mature ECs also regulate the behavior of smooth muscle cells [69].

The glycosaminoglycans Hep and heparan sulfate are involved not only in the efficient interaction of heparin-binding growth factors with their receptors in cells, but also in the storage and stabilization of GFs [30,63]. As the Hep-containing microbeads released only 1.3-fold more FGF-2 than the Alg/HSA microbeads (Figure 5A), no noticeable difference in the cell proliferation was expected. However, HUVEC proliferation in the Alg/HSA/Hep and Alg/HSA/HepII samples remained almost constant (approximately 80% of the EGMfull medium) from the day 1, while the relative cell index in the Alg/HSA and Alg samples decreased over time (Figure 7A). The cells also reached confluence in poor EGMm medium only when the Hep-containing microbeads were present (Figure 8). Furthermore, the Hep-containing microbeads were the only microbeads to release VEGF, which prevented cell detachment/death until day 5 (Figure 7B and Figure S7D’). These findings imply that Hep, when complexed with FGF-2 or VEGF, plays an important role in maintaining GF bioactivity under culture conditions.

The presence of Hep in scaffold/delivery systems can prolong GF/chemokine release from days to weeks. This outcome is very important, since a prolonged release of FGF-2 or VEGF supported in vitro ingrowth of adipose tissue-derived stem cells and their differentiation towards smooth muscle cells, as well as attachment of ECs on the pericardium surface [70], improved in vivo long-term colonization of grafts with ECs and smooth muscle cells with excellent graft patency after 18 months [71], or stimulated intensive in vivo vascularization of polylactide scaffolds within four weeks [72]. Furthermore, a long-term CXCL12 release prolonged islet/beta cell graft function without systemic immunosuppression [28,32]. The proposed composition of the high-G Alg microbeads enabled modulation of the long-term protein release in vitro for at least one month (Figure 4 and Figure 5) and provided promising potential for the effects of the tested CXCL12, FGF-2, and VEGF in cell stimulation under in vivo conditions. For example, the VEGF release profile may be beneficial, as it has been demonstrated that the application of a higher concentration of VEGF for a short period followed by lower concentrations is more effective for stimulating neovascularization [45,51].

Literature data suggest that no generally applicable principles for the release of proteins from Alg hydrogels can be easily established. Besides the surface net charge of proteins and other protein characteristics that determine protein/Alg interactions, the release of GFs appears to depend on the character of the hydrogel network, resulting from the content of G-units, the molecular weight, the crosslinking procedure used (i.e., internal or external gelation), or the type of divalent cations and their concentrations. The rate of protein release from Alg hydrogels can also be modulated by the change in pH or ionic strength in the release/surrounding medium [58], however, such conditions can rarely be met in vivo. The presented approach represents a simple way to enhance the release of proteins with higher p*I*, such as proteins used under this study, from high-G Alg hydrogel matrices without the need for chemical modification of the carrier. Complexation of positively charged proteins with polyanions can limit subsequent protein/Alg binding. Of the candidates appropriate as polyanions from the application perspective, the use of Hep appears to be beneficial also because of its widespread clinical use. High-G Alg matrices can be more preferred in TE applications with requirements for increased mechanical stiffness (e.g., bone TE applications) or hydrogel stability under physiological conditions such as cell encapsulation [73].

## 3. Materials and Methods

### 3.1. Materials

Human serum albumin (HSA), bovine serum albumin (BSA), and heparin (Hep) were obtained from Sigma-Aldrich (Prague, Czech Republic). Recombinant human stromal derived factor-1 alpha (CXCL12, CN 300-28A), fibroblast growth factor-2 (FGF-2, CN 100-18C), and vascular endothelial growth factor (VEGF165, CN 100-20) were purchased from PeproTech EC Ltd. (London, UK). Ultrapure low viscosity sodium alginate (UPLVG Alg) with a high content of guluronic acid unit (63% G-unit, determined by ^1^H-NMR analysis as shown in Figure S1, Table S1, see Supplementary Information, SI), viscosity of 20–200 mPa⋅s and weight average molecular weight of 75–250 kDa (as provided by the manufacturer) was purchased from NovaMatrix (Sandvika, Norway). The residual water content was 17 wt. %, as determined by gravimetry.

The 11-aminoundodecane-1-thiol hydrochloride, used for the preparation of functionalized surface plasmon resonance chips, was synthesized according to the modified literature procedure [74,75] in our laboratory (for details see the SI, Part S1.1).

Phosphate-buffered saline (PBS), 2-(N-morpholino) ethanesulfonic acid (MES) and sodium azide (NaN_3_) were purchased from Sigma-Aldrich (Prague, Czech Republic). The chemicals NaCl, BaCl_2_⋅2H_2_O, and CaCl_2_⋅2H_2_O were purchased from mikroCHEM (Pezinok, Slovakia). Milli-Q water was used for the preparation of all solutions. Alg solutions were filter-sterilized using syringe filters (0.22 µm, LOT-20150001, TPP, Trasadingen, Switzerland). The gelling and washing solutions were sterilized using bottle-top filters (0.2 µm, Filtropur BT50, LOT-60U1011, Sarstedt AG & Co. KG, Sarstedt, Germany).

### 3.2. Preparation of the Microbeads

The Alg microbeads were prepared by air-stripping the Alg solution in PBS into a gelling solution (1 mM BaCl_2_ and 50 mM CaCl_2_ in 0.9 wt. % NaCl solution, pH 7.4) followed by subsequent rinsing with a washing solution (2 mM CaCl_2_ in 0.9 wt. % NaCl_,_ pH 7.4) under sterile conditions according to the literature [36,37,60]. A more detailed description is presented in the SI (Part S1.2). The composition of Alg solutions used for microbead preparation is presented in Table 1.

**Table 1:** Composition of mixtures used for microbead preparation.

Sample Abbreviation	Alginate	Protein ^a^	HSA	Heparin
	(wt. %)			
Alg ^b^	1.3	0.003	-	-
Alg/HSA ^b^	1.3	0.003	0.01	-
Alg/Hep ^c^	1.3	0.003	-	0.03
Alg/HSA/Hep ^b^	1.3	0.003	0.01	0.03
Alg/HSA/Hep II ^b^	1.3	0.003	0.01	0.06

^a^ Protein-CXCL12, VEGF, FGF-2. ^b^ Microbeads prepared with CXCL12, VEGF, or FGF-2. ^c^ Microbeads prepared with only CXCL12.

### 3.3. Characterization of the Microbeads

The microbeads used for testing the size and compression resistance were stored in the washing solution after preparation at 6 °C until the use.

*Optical microscopy.* The size of the Alg microbeads was determined using an optical microscope (OPTIKA Microscopes B-383 PL, Kvant s.r.o., Bratislava, Slovakia) equipped with a CCD camera (Motic Moticam 1SP 1.3 MP) and Motic Images Plus 2.0 software. The determination of the microbead size was performed for 20 microbeads from each type, and was expressed as the average value ± standard deviation.

*Compression resistance*. A texture analyzer (TA-XT2i, Stable Micro Systems, Godalming, UK) equipped with a mobile probe and Texture Expert Exceed 2.64 software was used to determine the mechanical resistance of the prepared Alg microbeads in compression mode. The bursting force measurement was performed up to 95% deformation at a compression speed of 0.5 mm/s. Twenty to thirty microbeads were placed on a glass slide without removing the storage solution. Individual microbeads were placed under the probe, and the excess of the storage solution was carefully wiped off before measurement. The compression resistance of the Alg microbeads was evaluated as the force required for 70% deformation, expressed in g/microbead.

*Confocal laser scanning microscopy*. The distribution of HSA and Hep within the Alg microbeads was observed with an Olympus FV1000 confocal laser scanning microscope. HSA and Hep were fluorescently labeled with TAMRA (Fisher Scientific Ltd., Pardubice, Czech Republic) and FITC (Sigma–Aldrich, Prague, Czech Republic), respectively, using standard protocols [76]. Alg microbeads containing fluorescently labeled HSA and Hep were prepared as described in Section 2.2.

### 3.4. Evaluation of Interactions between Hep, HSA and Proteins with Alg Matrix

*Isothermal titration calorimetry (ITC)*. ITC was carried out at 25 °C on a MicroCal ITC200 (Malvern Panalytical Ltd., Malvern, UK) through a series of one to three subsequent titrations. Each titration was performed by 0.2-µL injection, followed by nineteen 2-µL injections. The series of raw titration data were then combined using ConCat32 software (Malvern Panalytical Ltd., Malvern, UK). Additionally, the titrant solutions were titrated into solvent, and the data were corrected to the corresponding heats of the dilution. The polymers used, i.e., Alg, HSA and Hep, were studied for their ability to bind a model protein lysozyme (Lz) in PBS by (i) direct titrations of the polymer to Lz or by (ii) displacement assays, when the polymer was titrated to Lz already complexed with another polymer. Global fitting of the isotherms for each system was carried out with AFFINImeter software ver. 1.2.3. (S4SD-AFFINImeter, Santiago de Compostela. Spain). From the fit obtained, the affinity constant, *K*_a_ (M^−1^), stoichiometry, *n* (number of carrier molecules per one Lz molecule), binding free energy (Δ*G*), binding enthalpy (Δ*H*) and binding entropy (Δ*S*) were calculated for 1 mol of titrant (i.e., Hep or HSA). A detailed description of each titration experiment is presented in the SI (Part S1.3).

*Surface plasmon resonance spectroscopy (SPR).* SPR analysis was performed on gold-coated SPR chips (Institute of Photonics and Electronics, Academy of Sciences of the Czech Republic, Prague, Czech Republic) modified (i) with a self-assembled monolayer (SAM) of 11-aminoundodecane-1-thiol hydrochloride (synthesized in our laboratory), which served as an anchoring layer, and then subsequently (ii) with a covalently attached Alg layer according to Pop-Georgievski et al. [77], which was crosslinked by Ba^2+^ and Ca^2+^ cations. The detailed conditions of the synthesis of 11-aminoundodecane-1-thiol hydrochloride, the SAM preparation and Alg coating are presented in the SI (Parts S1.1 and S1.4). The presence and thickness of the SAM and Alg layer on SPR chips were verified by XPS analysis and spectroscopic ellipsometry (SI, Part S2.2, Figures S2 and S3). The details of the methods used for these measurements are presented in the SI (Part S1.5).

Interactions between proteins and a crosslinked Alg layer deposited on an SPR chip were monitored as a shift in the resonance wavelength (Δ*λ*_res_) using a custom-built SPR sensor (Institute of Photonics and Electronics, Academy of Sciences of the Czech Republic, Prague, Czech Republic) at 25 °C. The modified SPR chip (maintained in H_2_O before use) was placed in the SPR instrument and equilibrated in PBS for 30 min. Then, solutions of CXCL12, VEGF, and FGF-2 (1 µg/mL), Hep (10 µg/mL) or HSA (3.6 µg/mL) and a CXCL12/Hep mixture in PBS and PBS as a control were pumped for 1 h into the SPR flow cell in independent channels at a flow rate of 25 µL/min.

### 3.5. Protein Release Studies

An accurately weighed amount of Alg microbeads (approximately 100 mg) with different compositions (see Table 1) was incubated with 1 mL of the releasing solution, i.e., PBS with the addition of 0.1 wt. % BSA and 0.02 wt. % NaN_3_, in 1.5-mL Eppendorf vials in a vertical rotator under slow agitation at 37 °C. Details on the sample preparation are given in the SI (Part S1.6). At predetermined times, i.e., 1, 2, 4, 8, 24, 48, 144, 213, 360, and 672 h, the releasing solution was removed, and fresh solution was added. The removed release solutions were divided into aliquots and stored at −80 °C prior to analysis by the enzyme-linked immunosorbent assay (ELISA). For all the studies, we used low-binding Eppendorf vials.

The amounts of the released CXCL12, FGF-2, and VEGF were quantified with the following ELISA kits according to each manufacturer’s instructions: human CXCL12/SDF-1 DuoSet ELISA and DuoSet ELISA Ancillary Reagent Kit 2 (DY350-05, DY008, R&D Systems, BioTech Ltd., Prague, Czech Republic), human FGF-basic Standard ABTS ELISA Development Kit and ABTS ELISA Buffer Kit (900-K08, 900-K00, PeproTech EC Ltd., London, UK), and human VEGF ELISA kit (KHG0112, Invitrogen, Fisher Scientific Ltd., Pardubice, Czech Republic).

### 3.6. In Vitro Studies

*Preparation of sample eluates*: An amount of microbeads weighing 100 mg was incubated with a mixture of PBS and 0.1 wt. % BSA for 5 days (1 mL) under sterile conditions. Then the releasing medium was withdrawn and stored in aliquots at −80 °C prior to use.

#### 3.6.1. Bioactivity of CXCL12 Released from Alg Microbeads

The bioactivity of CXCL12 released from the Alg microbeads was assessed by a Boyden chamber-based cell migration assay. The details of the experimental conditions are presented in the SI (Part S1.7). Briefly, Jurkat cells (a human immortalized T lymphoma cell line) were cultivated in an RPMI medium with 10% fetal bovine serum (FS) until passages three to ten. Then, using a 24-well plate, 10^5^ cells in 100 µL of RPMI cultivation medium with no serum were seeded into Millicell hanging cell culture inserts (5-µm pore size). The bottom compartment of each well was filled with 900 µL of the cultivation media and (i) 100 µL of the sample from the release experiments (see Section 2.4), (ii) 100 µL of PBS containing free CXCL12 (the final CXCL12 concentrations were 10, 25, or 50 ng/mL) as positive controls, or (iii) 100 µL of PBS as a negative control. The cultivation plates were incubated in 5% CO_2_ at 37 °C for 2 h, and then, the migrated cells were centrifuged, resuspended in 30 µL of PBS, and counted with a Bürker chamber. Cell migration was expressed as the % of the negative control, which was set as 100%.

#### 3.6.2. Bioactivity of VEGF and FGF-2 Released from Alg Microbeads

*Real-time evaluation of cell adhesion and growth*. Adhesion and proliferation of human umbilical vein endothelial cells (HUVECs) were evaluated using a xCELLigence real-time cell analyzer SP (ACEA Biosciences, Inc., San Diego, CA, USA). First, 160 µL of specific cell culture medium (see Table 2) was added into each well of an Agilent E-plate 96 PET plate (Accela Ltd., Prague, Czech Republic), and the chambers were calibrated. Then, 20 µL of the PBS solution containing VEGF or FGF-2 after the 5-day release from the microbeads, PBS (as a negative control), or cultivation medium (as a positive control) was added. Finally, 20 µL of the HUVECs suspension was added to each well. The total volume of the culture medium was 200 µL per well, and the final HUVEC seeding density (passages 3–4) was 3000 cells per well. A special cell cultivation medium was used for each tested group. A cultivation medium (C-22111, PromoCell GmbH, Biomedica CS Ltd., Prague, Czech Republic) with 2% FS and containing all growth factors (denoted EGMfull) was used for a positive control, and a cultivation medium with 2% FS without all growth factors except for epidermal growth factor (denoted EGM weak, EGMw) was used for sample testing and for a negative control, respectively. The composition of each culture medium is specified in Table 2. The electrical resistance, expressed as a cell index reflecting cell number and cell spreading, was measured on living cells every 15 min for 6 days (VEGF) or 7 days (FGF-2). The effect of VEGF or FGF-2 on the cell index was evaluated at different time intervals.

**Table 2:** Composition of each cultivation medium used for xCELLigence real-time cell analysis (RTCA) and for immunofluorescence staining.

Experiment	Sample	Medium Abbreviation	Composition of the Cultivation Medium *
xCELLigence RTCA	Positive control	EGMfull (C+)	2% of FS, EGF, Hep, ascorbic acid, hydrocortisone, 1% of ABAM, IGF, VEGF, FGF-2 (200 µL/well)
	VEGF microbeads	EGMw	2.2% of FS, EGF, Hep, ascorbic acid, hydrocortisone, 1% of ABAM (180 µL/well); 20 µL of the microbead eluate in PBS/well
	FGF-2 microbeads	EGMw	2.2% of FS, EGF, Hep, ascorbic acid, hydrocortisone, 1% of ABAM (180 µL/well); 20 µL of the microbead eluate in PBS/well
	Negative control	EGMw (C-)	2.2% of FS, EGF, Hep, ascorbic acid, hydrocortisone, 1% of ABAM (180 µL/well); 20 µL of PBS/well
Immuno-fluorescence staining	Positive control	EGMfull (C+)	2% of FS, Hep, ascorbic acid, hydrocortisone, 1% of ABAM, IGF, EGF, VEGF, FGF-2, (1000 µL/well)
	VEGF microbeads	EGMm	2.2% of FS, Hep, ascorbic acid, hydrocortisone, 1% of ABAM, IGF, EGF, 1/4 of the standard FGF-2 concentration (800 µL/well); 200 µL of the microbeads in EGMm/well
	Negative control (for VEGF)	EGMm (C-)	2.2% of FS, Hep, ascorbic acid, hydrocortisone, 1% of ABAM, IGF, EGF, 1/4 of the standard FGF-2 concentration (1000 µL/well);
	FGF-2 microbeads	EGMm	2.2% of FS, Hep, ascorbic acid, hydrocortisone, 1% of ABAM, IGF, EGF, 1/4 of the standard VEGF concentration (800 µL/well); 200 µL of the microbeads in EGMm/well
	Negative control (for FGF)	EGMm (C-)	2.2% of FS, Hep, ascorbic acid, hydrocortisone, 1% of ABAM, IGF, EGF, 1/4 of the standard VEGF concentration (1000 µL/well);

EGMw-“weak” EGM; EGMm-“moderate” EGM, ABAM–antibiotic-antimycotic solution. * Unless otherwise stated, the concentrations of the particular components were used according to the manufacturer’s protocol.

*Immunofluorescence staining of CD31, von Willebrand factor and VE-cadherin***.** HUVECs (passage 3) were seeded at a density of 30 000 cells/well in a 24-well glass-bottom plate (P24-1.5H-N, Cellvis, Mountain View, CA, USA) and cultured for 5 days in EGMm or EGMfull media (Table 2) in the presence of Alg-based microbeads loaded with VEGF or FGF-2. During this period, the cultivation medium was not changed. EGMfull medium (C-22111, PromoCell GmbH, Biomedica CS Ltd., Prague, Czech Republic) was used as a positive control, and cultivation medium with 2.2% FS containing only a limited number of growth factors, i.e., IGF, EGF, and a 1/4 of the standard FGF-2 or VEGF concentrations according to manufacturer’s protocols (denoted EGM moderate, EGMm) was used for sample testing (see Table 2 for the composition of each cultivation medium). EGMm allows slow growth of HUVECs and thus enabled us to distinguish the effect of the released growth factors on the cell growth and differentiation.

On day 5, the cells were fixed according to the standard protocol, and immunohistochemically stained by incubation with specific antibodies against platelet endothelial cell adhesion molecule-1 (CD31), VE-cadherin and von Willebrand factor, and then the cells were incubated with specific fluorescence labels. After washing the stained samples twice in PBS, images were taken using an Olympus IX71 epifluorescence microscope equipped with a DP80 digital camera at the same exposure time. The intensity of the immunofluorescence staining was evaluated using ImageJ software, and the intensity per image was measured and normalized per cell. Details of these procedures are presented in the SI (Part S1.8).

*Statistical analysis* was performed using a one-way analysis of variance (ANOVA) with a Student–Newman–Keuls test. The results are reported as mean ± SD.

## 4. Conclusions

This study reports on (i) preventing the interactions between an anionic high-G Alg hydrogel matrix and therapeutic proteins with a high p*I*, such as CXCL12, FGF-2, and the p*I* close to physiological pH, i.e., VEGF, to achieve prolonged protein release and (ii) in vitro bioactivity of the released proteins. The proposed approach consists of complexation of heparin-binding proteins with Hep as a polyanion to shield binding sites on proteins prior to their addition to the Alg solution.

Complexation with Hep reduced subsequent interactions of proteins with high-G Alg, as shown in the ITC and SPR model experiments. Accordingly, shielding the protein/Alg interactions allowed us to modulate protein release from the microbeads for 4 weeks. The presence of Hep affected the release of CXCL12, FGF-2, and VEGF in different ways. The shielding was most effective in the case of CXCL12, with the highest p*I* of 10.17, which is itself retained in the high-G Alg matrix. Alg/HSA/Hep microbeads released 500-fold and six-fold more CXCL12 and FGF-2, respectively, than pure Alg microbeads, whereas the release of VEGF with the p*I* of 7.02 was slower than the release from the Alg microbeads, but still uniquely with the zero-order kinetic profile. The released proteins retained their in vitro bioactivity. CXCL12 stimulated the migration of T lymphocytes, and FGF-2 and VEGF stimulated proliferation and maturation of HUVECs in nutrient-deprived culture media. Hep also intensified the biological efficiency of the released GFs. Only Hep-containing microbeads releasing FGF-2 or VEGF promoted cell proliferation, leading to well-developed confluent monolayers of HUVECs.

Prolonged modulated protein release in vitro for one month provides a promising potential for the long-term effects of biosignaling molecules in vivo. Different protein release profiles also suggest potential for combined protein release. The principle of preventing the protein/high-G Alg interactions can be applied not only to delivery systems in the form of particles, but also adopted with hydrogel bulk systems, porous scaffolds or 3D bioprinting methods.

## Data Availability

All the data are illustrated in the Figures and in the Supplementary data.

## Supplementary Materials

The following are available online at https://www.mdpi.com/article/10.3390/ijms222111666/s1, Supplementary materials present the details on the synthesis of 11-aminoundodecane-1-thiol hydrochloride and the experimental methods presented in the manuscript. Supplementary data are as follows: Figure S1: ^1^H-NMR spectra of UPLVG alginate in D_2_O, Table S1: Content of guluronic (G) and mannuronic (M) units in Alg chains (^1^H-NMR), Figure S2: High resolution S 2p core level XPS spectrum of 11NH2-C11-1SH SAM formed on SPR sensors, Figure S3: High resolution C 1s and N 1s core level XPS spectra of 11NH2-C11-1SH SAM and bound alginate films on SPR sensors, Figure S4: Characterization of microbeads prepared from pure UPLVG alginates, Figure S5: Isothermal titration calorimetry evaluation, Figure S6: Boyden chamber-based cell migration assay - optimization of the experimental set-up, Figure S7: Proliferation of HUVECs stimulated by FGF-2 and VEGF released from Alg, Alg/HSA, Alg/HSA/Hep and Alg/HSA/Hep II beads expressed as the cell index, Figure S8: Fluorescence intensities of immunostaining of CD31, VE-cadherin and von Willebrand factor in HUVECs cultured in the EGMm negative control, the EGMm medium with the beads releasing FGF-2 or VEGF and in the EGMfull positive control.
