# Supplementary material for: Complexation of CXCL12, FGF-2 and VEGF with Heparin Modulates the Protein Release from Alginate Microbeads
Source: Int J Mol Sci. 2021 Oct 28;22(21):11666. doi: 10.3390/ijms222111666 (PMC8583835; doi:10.3390/ijms222111666)
Supplement: Supplementary file 1 [file ijms-22-11666-s001.zip › ijms-1424117-supplementary.pdf]

Supplementary information for:

Complexation of CXCL12, FGF-2 and VEGF with heparin modulates the protein release from alginate microbeads.

Edyta Adrian [1,2], Dušana Treľová [3], Elena Filová [4], Marta Kumorek [1], Volodymyr Lobaz [1], Rafal Poreba [1], Olga Janoušková [1], Ognen Pop-Georgievski [1], Igor Lacík [3,5], Dana Kubies [1,*]

1. *Institute of Macromolecular Chemistry Czech Academy of Sciences, Heyrovsky sq.2, Prague 162 06, Czech Republic*
2. *Department of Chemical Engineering, University of Chemistry and Technology, Prague, Technicka 3, 166 28 Prague 6, Czech Republic*
3. *Polymer Institute of the Slovak Academy of Sciences, Dubravska cesta 9, 845 41 Bratislava, Slovakia*
4. *Department of Biomaterials and Tissue Engineering, Institute of Physiology of the Czech Academy of Sciences, Videnska 1083, 142 20 Prague, Czech Republic*
5. *Centre for Advanced Materials Application of the Slovak Academy of Sciences, Dubravska cesta 9, 845 11, Bratislava, Slovakia*

** Corresponding author: kubies@imc.cas.cz*

Table of Content

1 EXPERIMENTAL PART 3

1.1 Synthesis of 11-aminoundodecane-1-thiol hydrochloride (11NH2-C11-1SH) 3

1.2 Preparation of alginate microbeads 4

1.3 Isothermal titration calorimetry (ITC) 4

1.4 Preparation of samples for surface plasmon resonance spectroscopy (SPR) 5

1.5 Characterization of self-assembled monolayers and alginate films (for SPR analysis) 6

1.6 Protein release studies 6

1.7 *In vitro* bioactivity of CXCL12 released from microbeads evaluated by migration studies 7

1.8 *In vitro* bioactivity of VEGF and FGF-2 released from microbeads evaluated by immunofluorescence staining of CD31, von Willebrand factor and VE-cadherin 8

2 RESULTS AND FIGURES 9

2.1 Characterization of alginate 9

2.2 Characterization of self-assembled 11NH2-C11-1SH monolayers and Alg crosslinked films on SPR chips 10

2.3 Microbead characterization 12

2.4 Isothermal titration calorimetry (ITC) 13

2.5 Evaluation of *in vitro* bioactivity of proteins released from microbeads 14

3 REFERENCES 17

1 EXPERIMENTAL PART

1.1 Synthesis of 11-aminoundodecane-1-thiol hydrochloride (11NH2-C11-1SH)

Synthesis of 12NH2-C12-1SH was performed according to a modified literature procedure [4, 5] in several steps that are depicted in a reaction scheme presented below.

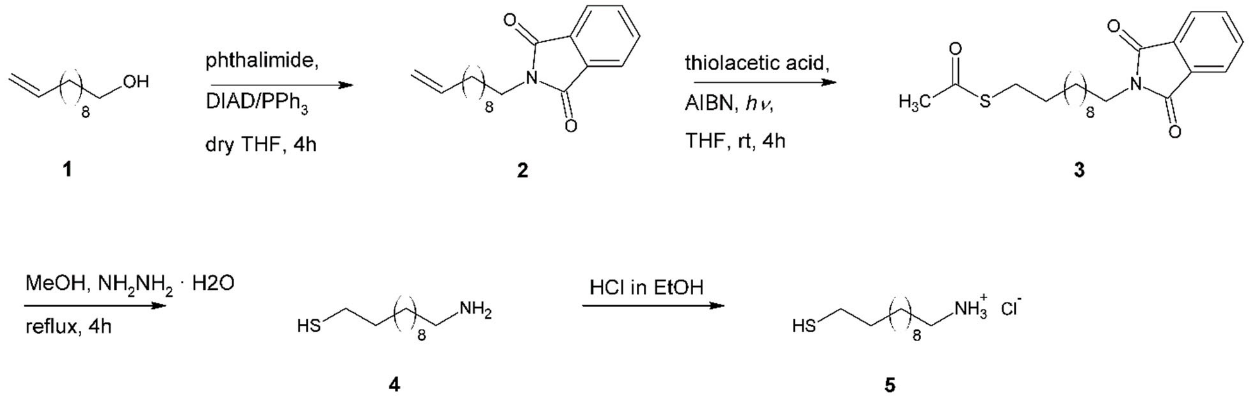

*Synthesis of 11-aminoundodecane-1-thiol hydrochloride: (**1**) 10-undecene-1-ol, **(2)** 2-(undec-10-en-1-yl)isoindoline-1,3-dione, **(3)** S-[10-(1,3-dioxo-1,3-dihydro-isoindol-2-yl)undecyl]ethanethioate, **(4)** 11-aminoundecane-1-thiol, **(5)** 11-aminoundodecane-1-thiol hydrochloride.*

Synthesis of 2-(undec-10-en-1-yl)isoindoline-1,3-dione (2):

10-undecene-1-ol (**1**) (11.8 ml, 58.1 mmol, 1.0 eq.), phthalimide (8.64 g, 58.1 mmol, 1.0 eq.) and triphenlyphosphine (15.42 g, 58.1 mmol, 1.0 eq) were suspended in anhydrous THF (60 ml). Then, diisopropylazodicarboxylate (DIAD) (12 ml, 58.1 mmol, 1.0 eq.) dissolved in dry THF (20 ml) was added to this suspension dropwise at 0 °C. The reaction mixture was warmed to room temperature and was stirred for 4h at room temperature. Afterwards, the solvent was removed using rotary evaporator and a cold diethyl ether (60 ml) was added, resulting in precipitation of triphenylphosphine oxide. The white solid (triphenylphosphine oxide) was filtered off and diethyl ether was removed from the reaction mixture using rotary evaporator, giving pale-yellow colored crystals. The crude product was purified by a column chromatography on silica gel (solvent hexane: EtOAc, 10:1 vol/vol) yielding 76 % of a clear crystalline product.

^{1}H NMR (300 MHz, DMSO-d6): δ 7.85 (m, 4H, Ar), 5.76 (m, 1H, -CH), 4.94 (m, 2H, CH_2), 3.55 (t, 2H, CH_2), 1.97 (q, 2H,CH_2) , 1.47 (tt, 2H, CH_2), 1.24 (m, 12H, CH_2).

Synthesis of S-[10-(1,3-dioxo-1,3-dihydro-isoindol-2-yl)undecyl] ethanethioate (3):

To the solution of the compound **2** (1.81 g, 6.0 mmol, 1 eq.) in dry THF (60 ml), thiolacetic acid (1.1 ml, 15.1 mmol, 2.5 eq.) and recrystallized azobis(isobutyronitrile) (AIBN) (82.7 mg) were added. The reaction mixture was stirred under argon atmosphere and UV source (254 nm) for 4 h. The solvent was removed

under reduced pressure to yield the crude product as a yellowish slurry. The final product **3** in form of white crystals was obtained after recrystallization from MeOH.

^{1}H NMR (300 MHz, DMSO-d6): δ 7.86 (m, 4H, Ar), 3.55 (t, 2H, CH_2), 2.77 (t, 2H, CH_2), 2.28 (s, 3H, CH_3), 1.57 (tt, 2H, CH_2), 1.47 (tt, 2H, CH_2), 1.25 (m, 14H, CH_2).

Synthesis of 11-aminoundecane-1-thiol (4) and 11-aminoundodecane-1-thiol hydrochloride (5)

To the solution of the compound **3** (1g, 2.7 mmol, 1eq.) in MeOH (50 ml), hydrazine monohydrate (0.53 g, 10.6 mmol, 4 eq.) was added and the mixture was refluxed for 4h. After the reaction mixture was cooled to room temperature, hydrochloric acid (20 ml of 2M solution) was added and stirring was continued for 20 minutes. The obtained precipitate of phthalhydrazide was filtered through a sintered glass filter and the filtrate was neutralized with 4M sodium hydroxide. Subsequently, the neutralized filtrate was extracted with diethyl ether (3x 40 ml). Collected organic phases were dried with $MgSO_4$ and diethyl ether was removed on rotary evaporator giving a white solid as the final product (**4**). To improve the stability of the final product, the compound **4** was quantitatively transformed into the hydrochloride form (**5**) using 1M ethanolic solution of hydrogen chloride at room temperature. After the ammonium salt formation, ethanol was removed on rotavapor and the final product was dried under vacuum.

^{1}H NMR (300 MHz, MeOD): δ 2.63 (t, 2H, CH_2), 2.49 (t, 2H, CH_2), 1.59 (m, 2H, CH_2), 1.47 (m, 2H, CH_2), 1.4 (m, 14H, CH_2).

1.2 Preparation of alginate microbeads

The alginate microbeads were prepared by air-stripping of the alginate solution in PBS (pH 7.4) into a gelling solution followed by subsequent rinsing with a washing solution under sterile conditions according to the literature [1-3]. Briefly, 0.75 ml of a 1.3 wt.% solution of alginate w/o and w/ the additives (HSA, Hep, protein) in PBS was air-stripped to 10 ml of the gelling solution (1 mM $BaCl_2$ and 50 mM $CaCl_2$ in 0.9 wt. % NaCl solution, pH 7.4) in the beaker; the collection time was 70-80s and the gelling time under gentle shaking was 6 mins. Then, the gelling solution was aspirated and microbeads were washed three times with 10 ml of 0.9% wt. NaCl solution containing 2 mM $CaCl_2$ solution, pH 7.4.

1.3 Isothermal titration calorimetry (ITC)

The solutions of alginate (0.033 mM, 0.5 mg/mL), HSA (0.35 mM, 22.9 mg/mL), lysozyme (0.35mM, 5 mg/mL), lysozyme with alginate (0.35 mM, 5 mg/mL and 0.033mM, 0.5 mg/mL, respectively) and lysozyme with HSA (0.35mM, 5 mg/mL and 0.35 mM, 22.9 mg/mL, respectively) were titrated with the solution of heparin (0.69 mM, 20 mg/mL).

The solutions of alginate (0.033 mM, 0.5 mg/mL), lysozyme (0.35mM, 5 mg/mL) and lysozyme with alginate (0.35mM, 5 mg/mL and 0.033 mM, 0.5 mg/mL, respectively) were titrated with the solution of HSA (0.69 mM, 45.7 mg/mL).

The solution of alginate (0.033 mM, 0.5 mg/mL) was titrated with the solution of lysozyme (1.53 mM, 22 mg/mL).

The binding parameters of lysozyme to alginate were determined in the so-called displacement assay, when lysozyme was complexed with a weaker binder, i.e. alginate or HSA, and the complex was then titrated with a stronger binder, i.e., HSA or heparin, respectively (Fig. 3). Thermodynamic parameters (K_a, ΔH) for binding of lysozyme with heparin and alginate were determined in a separate reference experiment after the global fit of two isotherms was performed, using a stoichiometric equilibria approach. The first isotherm (for a reference experiment) was always fitted with a simple binding model (one set of thermodynamic parameters) and the second isotherm (for a displacement assay) was fitted as a competitive binding model (two sets of thermodynamic parameters, one shared with the first isotherm, the second was independent). The independent set of thermodynamic parameters was used for the interaction of lysozyme with alginate. Several fittings cycles were performed for the systems heparin-HSA-lysozyme, heparin-alginate-lysozyme and HSA-alginate-lysozyme until the best fit was reached. Then thermodynamic parameters mutually satisfying all three systems were calculated.

1.4 Preparation of samples for surface plasmon resonance spectroscopy (SPR)

Preparation of 11NH2-C11-1SH self-assembly monolayer (SAM)

Prior to the surface modification, gold-coated SPR chips (Institute of Photonics and Electronics, Academy of Sciences of the Czech Republic) were rinsed with UV/HPLC spectroscopic grade ethanol, Milli-Q water, dried in N_2 stream and their surface was activated in UV-ozone cleaner (Jelight) for 20 min. Freshly activated chips were immediately immersed into a methanolic solution of 11-aminoundodecane-1-thiol hydrochloride (11NH2-C11-SH, 0.67 mg/ml, 2.7 mmol), and kept in the dark overnight. Following the SAM formation, SPR chips were rinsed with EtOH and Milli Q water and transferred into 0.05 M KOH solution (1:1 v/v, H_2O/EtOH) for 20 min to generate free amine -NH_2 groups at their surface. Finally, all the substrates were washed with Milli-Q water, EtOH and Milli-Q water, and dried in a N_2 stream. To prevent any surface contamination, they were kept under vacuum using a grease-free desiccator until use.

Deposition of the alginate layer

The alginate hydrogel layer was immobilized onto 11NH2-C11-SH-functionalized SPR chips using EDC/NHS chemistry according to Pop-Georgievski et al. [6]. Before the final coating of SPR chips, conditions of the immobilization were optimized. The conditions were adjusted to reach the immobilized Alg layer not thicker than 20 nm in dry state (spectroscopic ellipsometry evaluation). The presence of SAM and Alg on SPR chips were verified by XPS analysis (**Fig. SI2, Fig. SI3**).

11NH2-C11-SH-functionalized SPR chips were modified as follows: 0.4 wt % alginate solution in a 2-(N-morpholino)ethanesulfonic acid (MES) buffer (pH 5) was activated with EDC and NHS with the molar ratio uronic units:EDC:NHS = 1:20:20. The 12NH2-C12-SH-functionalized SPR chips were immersed in the

activated alginate solution for 2h at 25 °C, then the chips were rinsed thoroughly with Milli Q water and immersed into a 1mM $BaCl_2$/50 mM $CaCl_2$ co-solution for 30 min under mild shaking to crosslink the attached alginate chains. Final Alg-functionalized SPR chips were washed carefully with Milli Q water and kept swollen until the SPR analysis.

1.5 Characterization of self-assembled monolayers and alginate films (for SPR analysis)

Spectroscopic ellipsometry (SE)

J.A. Woollam M-2000X spectroscopic ellipsometer was used to measure the dry thickness of the SAM's and alginate films formed on SPR sensors. Ellipsometric data were obtained in the air at room temperature in the wavelength range λ = 245–1000 nm at angles of incidence of 60, 65, and 70°. The obtained data were analyzed with CompleteEASE software.

X-ray photoelectron spectroscopy (XPS)

Measurements were carried out with a K-Alpha$^+$ spectrometer (ThermoFisher Scientific, East Grinstead, UK). The samples were analyzed using a micro-focused, monochromated Al Kα X-ray source at an angle of incidence of 30° (measured from the surface) and an emission angle normal to the surface. The kinetic energy of the electrons was measured using a 180° hemispherical energy analyzer operated in the constant analyzer energy mode (CAE) at 200 eV and 50 eV pass energy for the survey and high-resolution spectra respectively. High-resolution Au 4f, S2p, C 1s, N 1s and O 1s core level and survey spectra were measured on layers formed on SPR sensors. Spectral resolutions of 0.1 and 1.0 eV were used for the high-resolution and survey spectra, respectively. All reported XPS spectra are averages of the twenty individual measurements referenced to the C1s peak of hydrocarbons at 285.0 eV. Data acquisition and processing were performed using Thermo Advantage software. The XPS spectra were fitted with Voigt profiles obtained by convolving Lorentzian and Gaussian functions. The analyzer transmission function, Scofield sensitivity factors, and effective attenuation lengths (EALs) for photoelectrons were applied for quantification. EALs were calculated using the standard TPP-2M formalism. The BE scale was controlled by the well-known position of the photoelectron C-C and C-H, C-O and C(=O)-O C 1s peaks of polyethylene terephthalate and Cu 2p, Ag 3d, and Au 4f peaks of metallic Cu, Ag and Au, respectively. The BE uncertainty of the reported measurements and analysis is in the range of ±0.2 eV.

1.6 Protein release studies

Samples preparation: 0.3 ml of the alginate microbeads of different compositions (Table 1) were pipetted using tips with a cut end into 1.5 ml Eppendorf tubes with known weights. The microbeads were let to settle down, an excess of the washing solution was carefully aspirated off, and the Eppendorf tubes were reweighted to determine weight of the microbeads. (Note: the weight of wet microbeads after aspiration of the washing solution from 1 mL of the microbead suspension was 433 mg.) Then, 1 ml of the PBS/0.1%

BSA/0.02% NaN_3 releasing solution was added into each Eppendorf tube. The closed Eppendorf tubes were then properly sealed with parafilm and placed in a vertical rotator under slow agitation at 37 °C.
At predetermined times, i.e., 1, 2, 4, 8, 24, 48, 144, 213, 360 and 672 h, the releasing medium was withdrawn and a fresh medium was added. The withdrawn release solutions were divided into aliquots and stored at - 80°C until the Enzyme Linked Immuno Sorbent Assay (ELISA). For all the studies, we used low-binding Eppendorf tubes.
The amount of the released CXCL12, FGF-2 and VEGF was quantified by ELISA using: human CXCL12/SDF-1 DuoSet ELISA and DuoSet ELISA Ancillary Reagent Kit 2 (DY350-05, DY008, R&D Systems), human FGF-basic Standard ABTS ELISA Development Kit and ABTS ELISA Buffer Kit (900-K08, 900-K00, PeproTech EC Ltd.) and using human VEGF ELISA kit (KHG0112, Invitrogen) according to the manufacturer's protocols. Before each ELISA evaluation, preliminary tests adjusting the sample dilution to reach a detection range for a particular ELISA set were performed.

1.7 *In vitro* bioactivity of CXCL12 released from microbeads evaluated by migration studies

Sample preparation: The known amount of the particular microbeads was incubated with PBS/0.1% BSA for 5 days, then the releasing medium was withdrawn and stored in aliquots at -80 °C prior the use.
The bioactivity of CXCL12 released from the alginate microbeads was assessed by a *Boyden chamber*-based cell migration assay. First, four cell lines, i.e., mouse macrophages cell line RAW 264.7, mouse macrophages cell line J774A.1, human T lymphoma cell line (Jurkat cells), and mouse mammary carcinoma cell line 4T1, were tested for expression of the cell membrane CDCR4 receptor by FACS analysis using intact or permeabilized cells (permeabilization: BD Cytofix/Cytoperm kit). The cells were labelled with anti CXCR4-FITC antibody (Bioss, **BioTech Ltd., Prague, Czech Republic**) for 30 min in dark at room temperature. Then, the cells were analysed using BD FACS Verse and Flow Jo software. As median of fluorescent intensity was the highest for Jurkat cells (Fig. SI 4A), these cells were used for further experiments.

The Jurkat cells were cultivated in a RPMI medium with 10% of fetal bovine serum (FS) added. Before each migration experiment, the cells were again tested for expression of the CDCR4 receptor and subsequently for the cell chemotaxis stimulated by a free CXCL12 added into the cultivation media. We used the cells from 3rd to 10th passage.

The chemotactic response of Jurkat cells was evaluated using a 24-well plate in a combination with polyethylene terephthalate Millicell hanging cell culture inserts with pore size of 5.0 µm (Sigma Aldrich, Prague, Czech Republic).) as follows: 10^5 cells in 100 µl of the RPMI cultivation medium without serum were seeded into each insert. Then, the bottom compartment of each well was filled with 900 µl of the cultivation media and a) 100 µl of the test sample from the release experiments (see Section 2.5), b) 100 µl of PBS containing free CXCL12 (final CXCL12 concentrations of 10, 25 or 50 ng/ml) as positive controls or c) 100 µl of PBS as a negative control; the total volume in a bottom chamber was 1 ml per well. The

cultivation plates were incubated in 5% CO_2 at 37 °C for 2 h. Then the inserts were removed and suspensions with the migrated cells from particular bottom compartments were transferred to Eppendorf tubes and centrifuged at 1500 rpm for 3 min. The supernatant was aspirated off, the cells were resuspended in 30 µl of PBS and the number of isolated cells was calculated using a Bürker chamber from eight different microscopic fields. Cell migration was expressed as a % to the negative control.

1.8 *In vitro* bioactivity of VEGF and FGF-2 released from microbeads evaluated by immunofluorescence staining of CD31, von Willebrand factor and VE-cadherin

The aliquots of frozen microbeads were resuspended in the cultivation media. HUVECs (the passage 3) were seeded at the density of 30 000 cell/well (in 100 µl) in a 24-well glass bottom plate (P24-1.5H-N, Cellvis, CA, U.S.A.) containing 700 µl of the cultivation media and 200 µl of the suspension of the Alg-based microbeads loaded with VEGF or FGF-2 in the cultivation media and cultured for 5 days. During this period, the cultivation medium was not changed. The EGMfull medium used for positive controls and the cultivation medium with 2.2 % FS containing only a limited number of growth factors (denoted as EGMm, moderate) was used for sample testing (see Table 2 for the composition of the cultivation media). The EGMm allows slow growth of HUVECs and thus enables to distinguish the effect of the released growth factors on cell growth and differentiation.

On day 5, the cells were fixed with 4% paraformaldehyde, permeabilized using a 1 wt.% BSA/0.1 wt.% Triton X-100 solution in PBS for 20 min, washed with PBS and treated with a 1 wt.% Tween 20, (Sigma-Aldrich, Prague, Czech Republic) for 20 min at RT. Then the samples were washed with PBS and incubated with mouse monoclonal antibody to CD31 (clone MEM-05, Cat. NO. 11-273-C100, Exbio, Prague, Czech Republic, dilution 1:200), polyclonal antibody to CD144 for detection of VE-Cadherin (purified IgG, produced in rabbit, Cat. No. AHP628Z, AbD Serotec, UK, dilution 1:200) and with anti-human von Willebrand factor IgG fraction of rabbit antiserum (F3520, SIGMA, dilution 1:200) overnight at 4-8 °C. After washing twice with PBS, the samples were incubated either with Alexa Fluor 488®-conjugated F(ab') fragment of goat anti-mouse IgG (H1L, Cat. No. A11017, dilution 1:1000; the CD31 detection) or Alexa Fluor 546® goat anti-rabbit IgG (H+L) (Cat. No. 11010, Thermofisher Scientific, Pardubice, Czech Republic, dilution 1:1000; the VE-Cadherin and von Willebrand factor detection) and DAPI (Sigma, 50 ng/mL; the nucleus detection) for 1 hour at RT. After washing twice in PBS, images were taken using an Olympus IX71 epifluorescence microscope equipped with a DP80 digital camera at the same exposure time. Intensity of the immunofluorescence staining was evaluated using ImageJ software. For all images, at the same threshold, the background was subtracted and the intensity per image was measured and normalized per cell.

Statistical analysis was performed using a one-way analysis of variance (ANOVA) with a Student–Newman–Keuls test to a significance level of $p < 0.05$. The results are reported as mean ± SD.

2 RESULTS AND FIGURES

2.1 Characterization of alginate

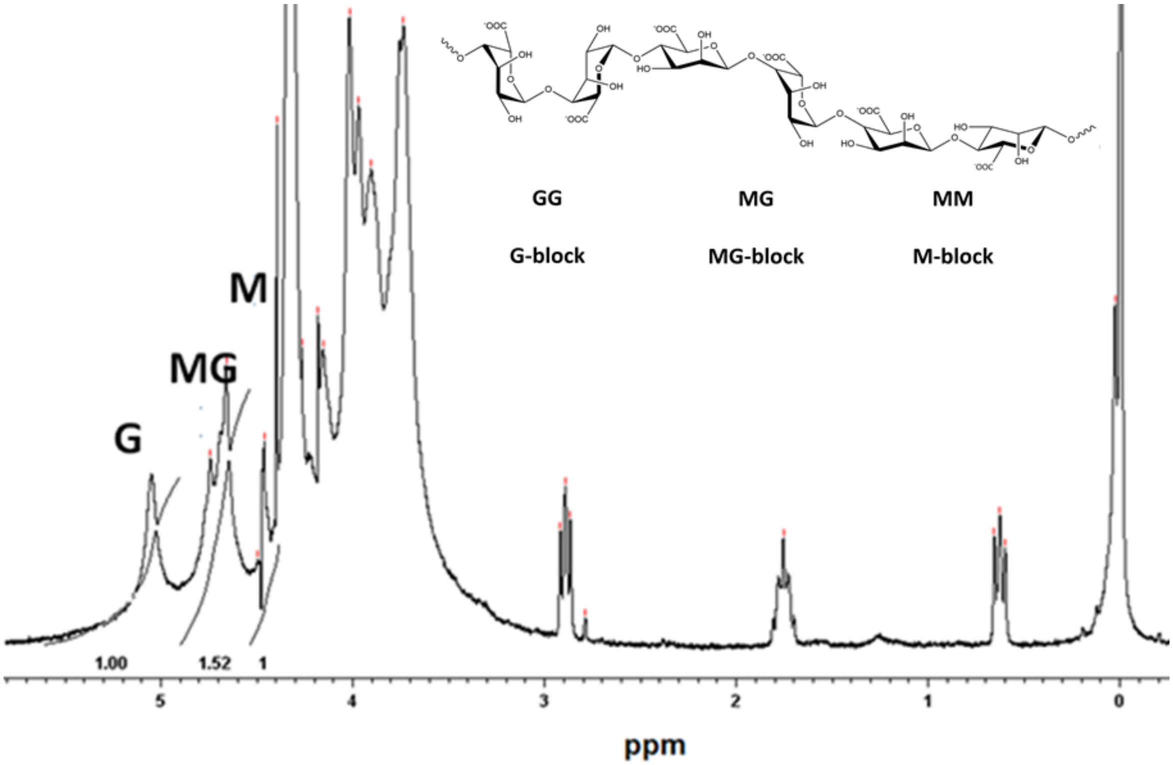

Figure S1 ^{1}H-NMR spectra of UPLVG alginate in D_2O

Table S1 Content of guluronic (G) and mannuronic (M) units in Alg chains (^{1}H-NMR).

Alginate	M/G	F_G	F_M	F_{GG}	F_{GM}	F_{MM}
UPLVG	0.59	0.63	0.37	0.38	0.25	0.12

2.2 Characterization of self-assembled 11NH2-C11-1SH monolayers and Alg crosslinked films on SPR chips

The thickness of the amino terminated SAM's and the subsequently bound alginate films were determined to be 1.6 and 12.0 nm, respectively. The SAM layer on the SPR chips showed the characteristic S 2p peak (composed of a spin split doublet S $2p_{3/2}$–S $2p_{1/2}$) of C-S-Au thiolate at 161.7 eV (**Figure S2**). The corresponding C 1s spectrum is characterized by the dominant C-C and C-H contribution at 285.0 eV accompanied by a shoulder at about 286 eV from C-N and C-S moieties of amine and thiolate groups (**Figure S3**). The N 1s spectrum of the SAM shows the characteristic contributions of amines at 399.9 eV and charged ammonium NH_3^+ groups at 401.8 eV. The immobilization of alginate on the surface is evidenced by the rise of the charged carboxylate COO^- and amide moieties in the C 1s spectrum at about 288.3 eV and the concomitant rise of amides C=O-NH in the N 1s spectrum (**Figure S3**). The concomitant SE and XPS data verified the formation of reference alginate films for studying the interactions of alginate films with various proteins.

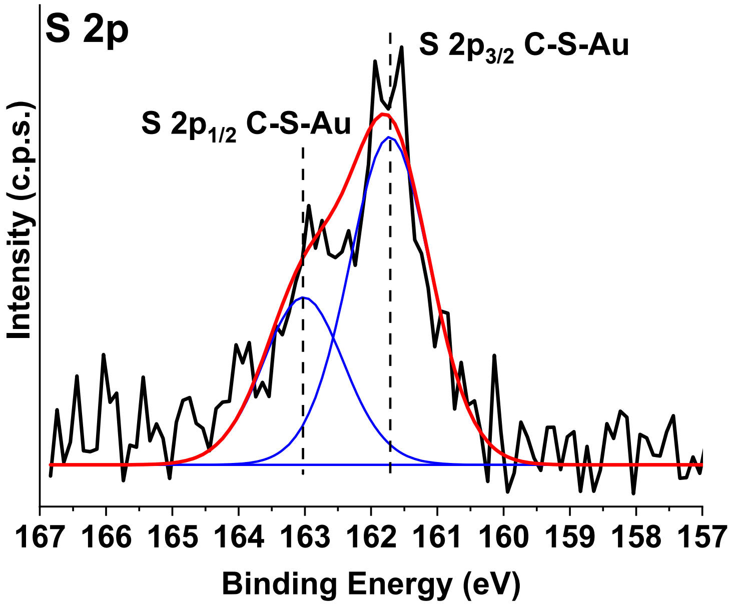

Figure S2 High resolution S 2p core level XPS spectrum of 11NH2-C11-1SH SAM formed on SPR sensors. Measured spectra (black) were fitted with individual contributions (blue) to obtain the resulting envelope (red).

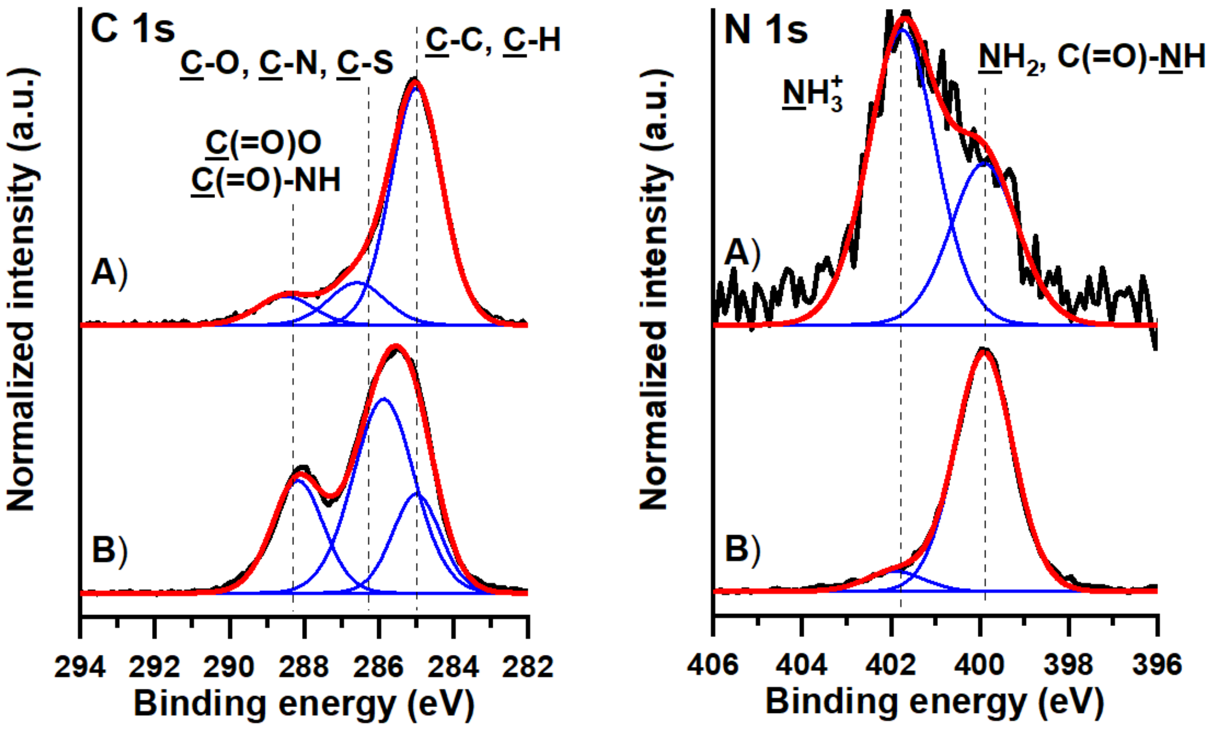

Figure S3 High resolution C 1s and N 1s core level XPS spectra of (A) 11NH2-C11-1SH SAM and (B) bound alginate films on SPR sensors. Measured spectra (black) were fitted with individual contributions (blue) to obtain the resulting envelope (red).

2.3 Microbead characterization

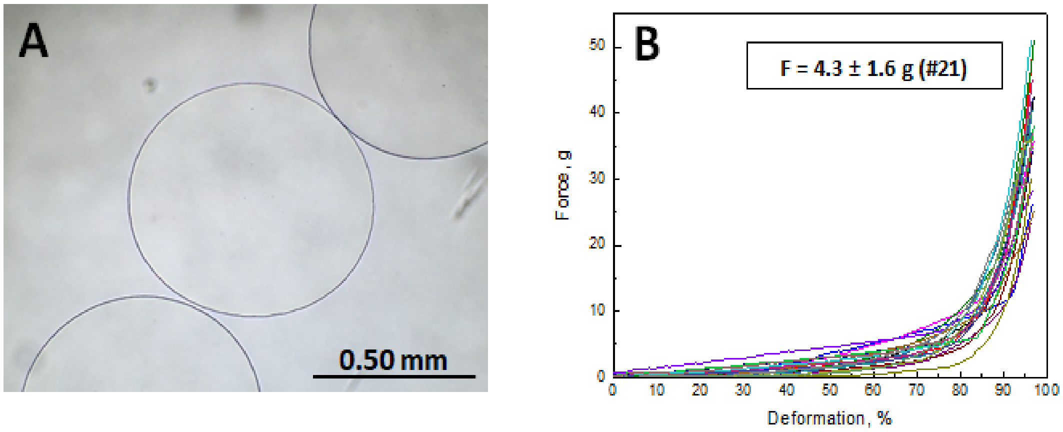

Figure S4 Characterization of microbeads prepared from the pure UPLVG alginates: A) A typical morphology of UPLVG microbeads (light microscopy); B) Compression curves for UPLVG beads showing the dependence of compression force (*F*, in grams) on the bead deformation; the value expresses the compression force needed for 70% of the bead deformation (n=21).

2.4 Isothermal titration calorimetry (ITC)

The dilution of a polymer solution as well as titration of one non-interacting polymers with another is always accompanied with a measurable heat, either released or absorbed. The heat originates from the perturbation of polymer equilibrium conformation upon dilution and from rearrangement of the solvation shell during the establishment of new equilibrium.

The carrier polymers (i.e., Alg, Hep, HSA) demonstrate no binding, however, the heat in range ±20 kJ/mol of injectant is absorbed or released during mixing of their solutions (**Figure S5A**). The titration isotherm of alginate with lysozyme (**Figure S5B**) shows extremely low heat of interaction (±2 kJ/mol) in comparison with the heat of the lysozyme dilution (which is in the range from 14 to 4 kJ/mol). Therefore, the isotherm on **Figure S5B** is considered erroneous and not applicable for the direct determination of thermodynamic parameters of the interaction.

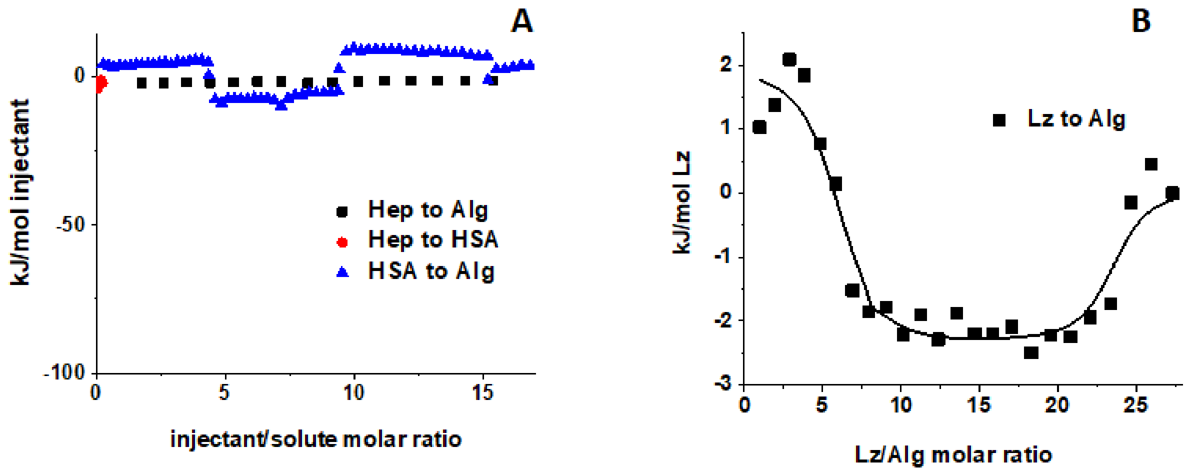

Figure S5 Isothermal titration calorimetry evaluation: A) Titration isotherms of carriers Alg, HSA and Hep polymers. B) Titration isotherm of Alg with lysozyme (Lz).

2.5 Evaluation of *in vitro* bioactivity of proteins released from microbeads

A

Cell type	The median of fluorescent intensity		
	State of cells		
	Non-labelled	Labelled - permeable	Labelled - intact
RAW 264.7	83.3	667	1254
J774A.1	92.1	828	1732
Jurkat cells	20.8	480	1846
4T1	47.6	480	308

B

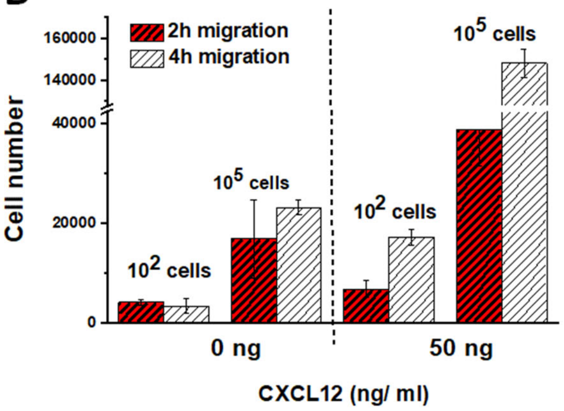

C

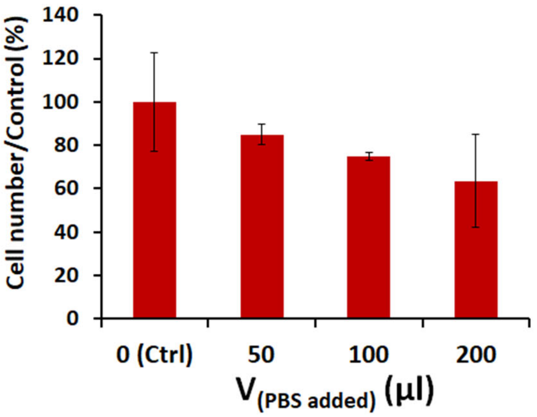

Figure S6 Boyden chamber-based cell migration assay - optimization of the experimental set-up*:* A) Selection of cell type: expression of the membrane CXCR4 receptor for CXCL12 by mouse macrophages cell line RAW 264.7, mouse macrophages cell line J774A.1, human T lymphoma cell line (Jurkat cells) and mouse mammary carcinoma cell line 4T1, (immunostaining, FACS analysis). All cell lines expressed the CXCR4 receptor on the cell membrane. The median of fluorescent intensity was the highest in Jurkat cells followed by J7, RAW and 4T1 cells. The permeabilization did not enhance the antibody binding, thus the epitope was well recognized by the anti CXCR4-FITC antibody; B) Effect of the number of seeded cells and migration time on the number of migrated Jurkat cells w/o stimulation by CXCL12 present in the cultivation medium; C) Effect of the volume of PBS added into the cultivation medium on the number of migrated cells (a total volume of medium was 1 ml).

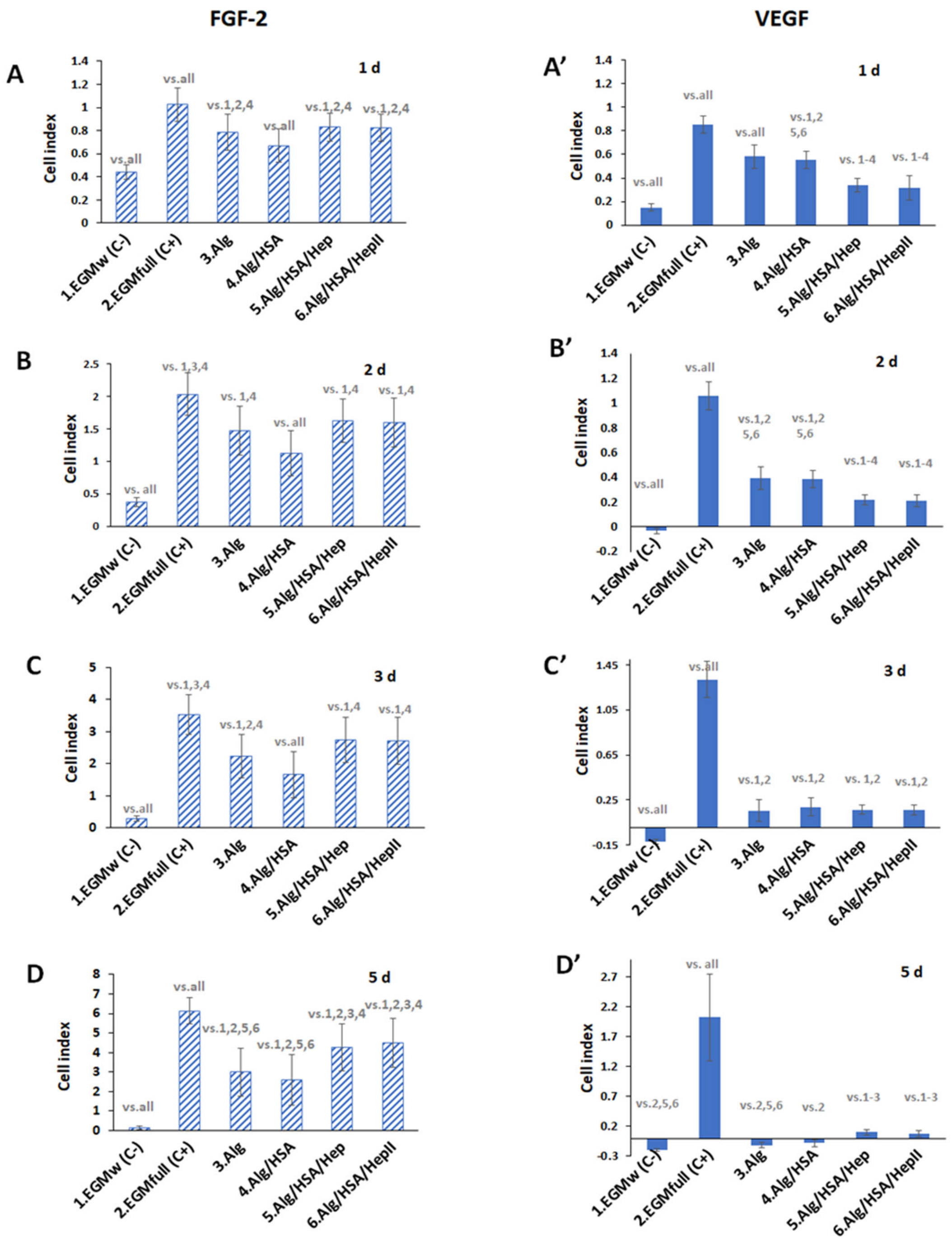

Figure S1 Proliferation of HUVECs stimulated by FGF-2 (A-D) and VEGF (A'-D') released from Alg, Alg/HSA, Alg/HSA/Hep and Alg/HSA/Hep II beads expressed as the cell index after 1 day (A, A'), 2 days (B, B'), 3 days (C, C') and after 5 days (D, D'). The cultivation medium without growth factor supplements, EGMw(C-), was used as a negative control and the cultivation medium fully supplemented with all growth factors, EGMfull(C+), was used as a positive control. (xCELLigence real-time cell analysis; the composition of each cultivation medium is presented in Table 2). The mean ± standard deviation was determined from 6 to 14 independent measurements. One-way analysis of variance and the Student–Newman–Keuls method were used for statistical analysis. A p value ≤ 0.05 was considered to be significant. Indicators above the bars show statistical differences compared to the samples identified with number indicated.

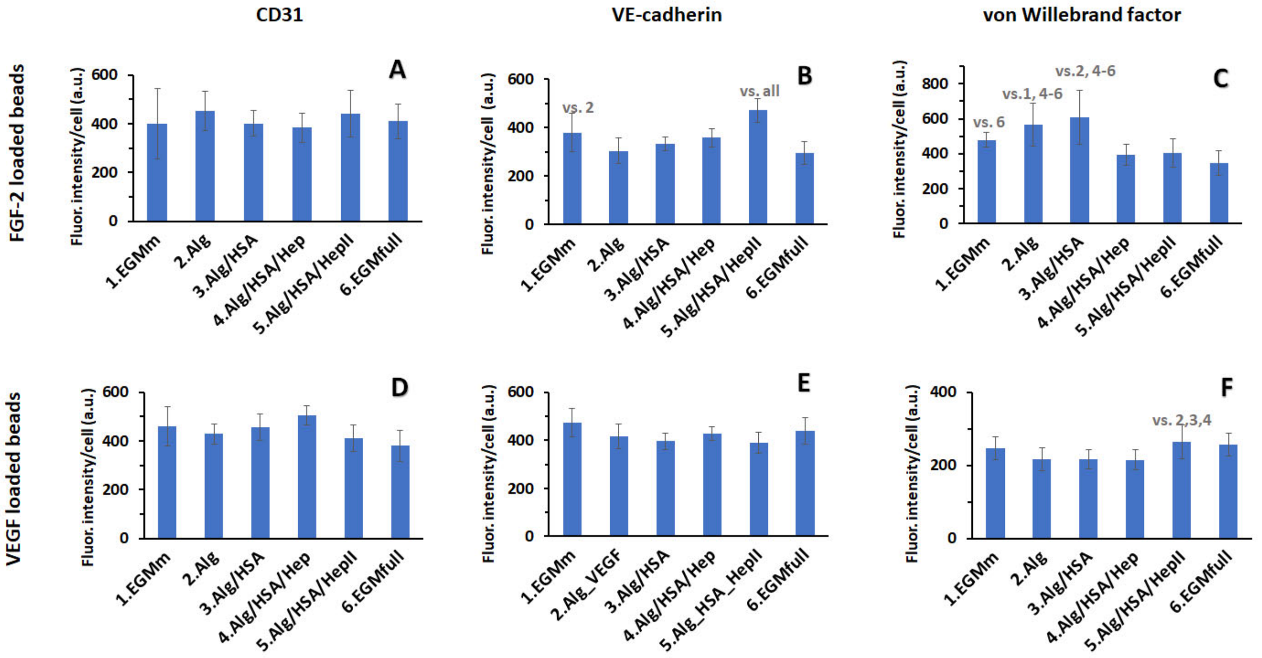

Figure S8 Fluorescence intensities of immunostaining of CD31 (A, D), VE-cadherin (B, E) and von Willebrand factor (C, E), normalized per cell, in HUVECs cultured in the EGMm negative control, EGMm medium with the beads releasing FGF-2 (A-C; upper raw) or VEGF (D-F; bottom raw), i.e., Alg, Alg/HSA, Alg/HSA/Hep, and Alg/HSA/HepII beads, and in the EGMfull positive control. Mean ± S.D., from 8-14 measurements, One Way Analysis of Variance, Student–Newman–Keuls method. P value ≤ 0.05 was considered significant, statistical differences among samples are marked above the bars compared to the samples of the same number.

3 REFERENCES

1. Y.A. Mørch, M. Qi, P.O.M. Gundersen, K. Formo, I. Lacik, G. Skjåk-Bræk, J. Oberholzer, B.L. Strand, Binding and leakage of barium in alginate microbeads, Journal of Biomedical Materials Research Part A, 100A (2012) 2939-2947.
2. M. Qi, Y. Mørch, I. Lacík, K. Formo, E. Marchese, Y. Wang, K.K. Danielson, K. Kinzer, S. Wang, B. Barbaro, G. Kolláriková, D. Chorvát Jr, D. Hunkeler, G. Skjåk-Bræk, J. Oberholzer, B.L. Strand, Survival of human islets in microbeads containing high guluronic acid alginate crosslinked with Ca2+ and Ba2+, Xenotransplantation, 19 (2012) 355-364.
3. M. Takacova, G. Hlouskova, M. Zatovicova, M. Benej, O. Sedlakova, J. Kopacek, J. Pastorek, I. Lacik, S. Pastorekova, Encapsulation of anti-carbonic anhydrase IX antibody in hydrogel microspheres for tumor targeting, Journal of Enzyme Inhibition and Medicinal Chemistry, 31 (2016) 110-118.
4. G. B. Sigal, M. Marksich, G. M. Whitesides - *J. Am. Chem. Soc.* 1998, 120, 14, 3464–3473
5. Debjyoti Bandyopadhyay, Deepali Prashar and Yan-Yeung Luk, *Chem. Commun.*, 2011,**47**, 6165-6167
6. O. Pop-Georgievski, D. Kubies, J. Zemek, N. Neykova, R. Demianchuk, E. Mazl Chanova, M. Slouf, M. Houska, F. Rypacek, *Beilstein J. Nanotechnol*., 2015, 6, 617-631.
